# Persistence of multiple patterns and intraspecific polymorphism in multi-species Müllerian communities of net-winged beetles

**DOI:** 10.1186/s12983-019-0335-8

**Published:** 2019-10-17

**Authors:** Matej Bocek, Dominik Kusy, Michal Motyka, Ladislav Bocak

**Affiliations:** Laboratory of Molecular Systematics, Faculty of Science, Palacky University, tr. 17. listopadu 50, 771 46 Olomouc, Czech Republic

**Keywords:** Müllerian mimicry, NextRAD, mtDNA, Phylogeny, Lycidae, New Guinea

## Abstract

**Background:**

In contrast to traditional models of purifying selection and a single aposematic signal in Müllerian complexes, some communities of unprofitable prey contain members with multiple aposematic patterns. Processes responsible for diversity in aposematic signaling are poorly understood and large multi-species communities are seldom considered.

**Results:**

We analyzed the phylogeny and aposematic patterns of closely related *Eniclases* net-winged beetles in New Guinea using mtDNA and nextRAD data. We suggest three clades of closely related and incompletely reproductively isolated lineages, detail the extent of polymorphism among *Eniclases*, and categorize their low-contrast aposematic patterns. The warning signal of *Eniclases* consists of body shape and color, with ambiguous color perception under some circumstances, i.e., when resting on the undersides of leaves. Field observations suggest that perception of the aposematic signal is affected by beetle behavior and environmental conditions. Local communities containing *Eniclases* consisted of 7–85 metriorrhynchine species assigned to 3–10 colour patterns.

**Conclusion:**

As a result, we suggest that under certain light conditions the aposematic colour signal is less apparent than the body shape in net-winged beetle communities. We document variable environmental factors in our study area and highly diverse multi-species communities of other net-winged beetles. Which implies dynamically changing community structure in space and time. Variable environmental conditions and diverse community composition are suggested to be favorable for the persistence of multiple aposematic patterns, imperfect mimics, and intraspecific polymorphism. Further research should identify the relative effect of these factors on purifying selection and the alleles which are responsible for phenotypic differences.

**Electronic supplementary material:**

The online version of this article (10.1186/s12983-019-0335-8) contains supplementary material, which is available to authorized users.

## Background

Müllerian mimicry is among the best-studied examples of evolution, yet some theoretical predictions stand in contrast with observed mimetic communities in nature [1–3]. Although the number of exhibited patterns should be quickly reduced by natural selection [4–6], we commonly observe high variation in mimetic signals in a single place, or intraspecific polymorphism [7–10]. Evidently, some factors must diminish predator learning of the association between the visual warning signal and negative stimulus produced by unprofitable prey, thereby decreasing the effectiveness of purifying selection. Inquiry into potential factors has included differences in unpalatability (quasi-Batesian mimicry), the effects of multimodal signals, environmental conditions and community structure [11–13]. Non-adaptive genetic constraints may correlate with observed long-term persistence of a high number of distinct aposematic patterns in one place [7, 14], where such constraints include the inability to produce pigments necessary for advergence to the dominant, most effective pattern, or considerable delays in pigment production [15, 16]. Mimics may differ in body structure such that only imperfect mimicry can be produced [2, 7, 13]. Differing interactions between genes and the environment among species may further prevent the dominance of a single signal in one community, for example, different melanization levels in response to a cold, humid climate in closely related *Cautires* beetles [17]. The origins and processes of imperfect mimicry have recently been reviewed [14], and in this study, we attempted to identify processes that may produce multi-pattern communities and counterbalance the hypothesized effects of selection for monomorphism in models of mimicry evolution [3, 4].

Net-winged beetles (Coleoptera: Lycidae) are considered Müllerian mimics, known for their unprofitability and aposematic coloration [18–20]. We focused on *Eniclases*, a trichaline genus of Metriorrhynchina from New Guinea [21, 22]. Having very closely related focal species, we expect that they do not differ substantially in the levels of their protection [7, 13, 16]. Patterns exhibited by trichalines are simple, combining two colors at most, namely, black and shades of yellow and orange in *Eniclases*. Unlike well-studied mimetic systems, few data are available regarding predation of *Eniclases*. The previous studies reported predators of net-winged beetles among birds, spiders, assassin bugs, and mantids [20].

**Fig. 1:**
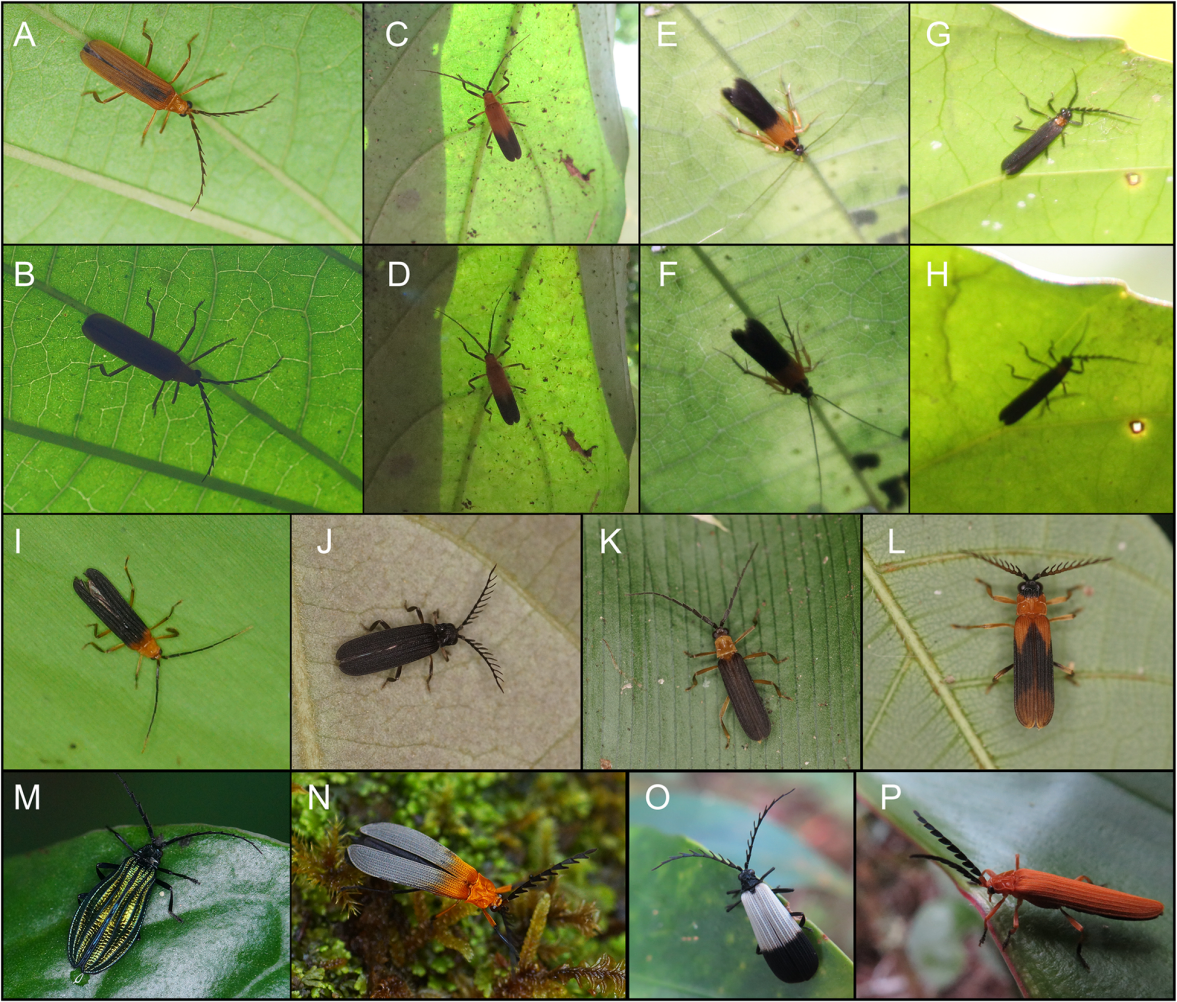
Trichalini and their co-mimics as observed in nature. (**a**–**h**) – the examples of internal and external contrast of the signal of an individual depending on light conditions (the upper photo taken with flash); (**a**–**b**) – Eniclases sp.; (**c**–**d**) – *Microtrichalus* sp.; (**e**–**f**) – unidentified moth; (**g**–**h**) – *Metriorrhynchus* sp. Co-mimics of *Eniclases*: (**i**, **k**) – *Microtrichalus* sp., (**j**, **l**) – *Metriorrhynchus* sp. The representatives of further aposematic patterns recorded in the region: (**m**) – *Diatrichalus aeneus* Bourgeois, (**n**) – *Cladophorus* sp., (**o**–**p**) – *Metriorrhynchus* sp.

**Fig. 2:**
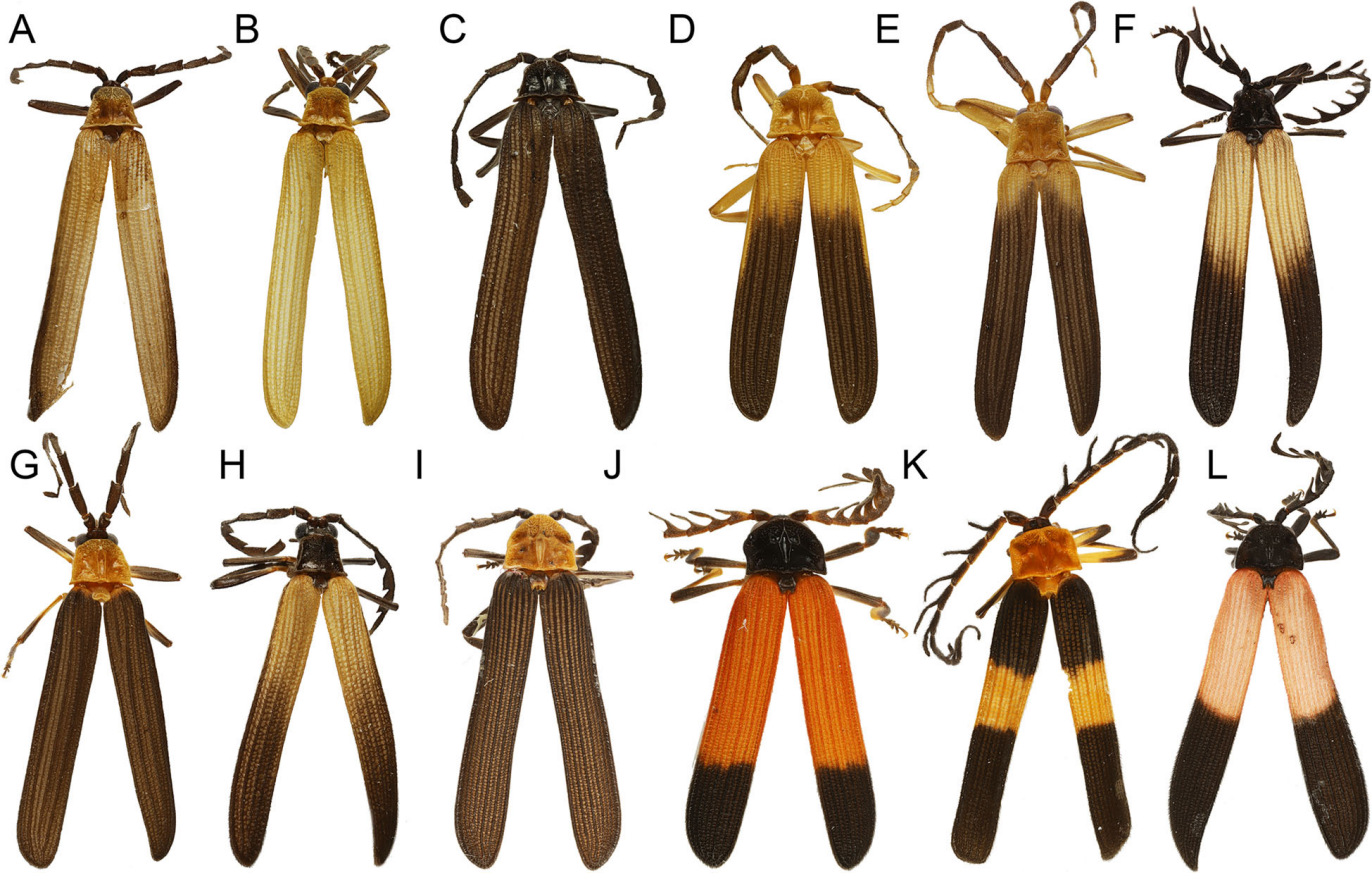
General appearance of trichaline co-mimics. (**a**–**h**) – *Microtrichalus* spp. Other aposematically coloured Metriorrhynchina from New Guinea. (**i**) – *Cautiromimus* sp., (**j**) – *Ditua* sp., (**k**) – *Carathrix* sp., (**l**) – *Metriorrhynchus* sp.

*Eniclases* belongs to the clade of Australian Metriorrhynchina, which dominates lycid communities in New Guinea [23, 24]. More than 300 Metriorrhynchina have been described from this region, but preliminary analyses of molecular data suggest even greater species diversity. Most Metriorrhynchina are brightly colored, with patterns combining high-contrast red and black areas, metallic blue or green coloration, various patches and bands, and examples of tri-colored elytra (Figs. 1, 2, [25]). In total, 36 species of *Eniclases* are known and their relationships with *Trichalus* and related genera (hereafter, trichaline genera) have been established using molecular data and morphology [23, 24]. In New Guinea, trichalines such as *Eniclases* and *Microtrichalus* dominate mimetic systems at low elevations but are uncommon at elevations > 1500 m [24]. All net-winged beetles depend on humid forest, remaining mostly inactive under forest canopies. As a result, their dispersal propensity is low and no metriorrhynchine species have been simultaneously recorded from landmasses separated by open sea [17].

Currently, mimicry literature is biased to butterflies, the best studied model group (e.g., [4, 10, 25], with limited representations of mimicry in other animals (e.g., [26–28]). Therefore, we focused on New Guinean Müllerian beetle communities containing *Eniclases* and their metriorrhynchine co-mimics. Net-winged beetles are known as unprofitable and aposematic, yet have only recently been studied [16, 18, 29] and the high diversity of aposematic patterns, and ecosystems in our study area provide a unique opportunity to investigate the pattern origins and composition of mimetic communities. We observed *Eniclases* in nature and investigated the New Guinean *Eniclases* and net-winged beetles communities to answer the following questions: Do the local communities contain species displaying multiple aposematic patterns or intraspecific polymorphism? Can the perception of aposematic signal differ under some conditions? How dissimilar are New Guinea net-winged beetle communities in species representation? We use RAD and mitochondrial DNA data to investigate if the phenotypic similarity is a result of relationships (aposematic signaling) or if the close similarity is encountered in unrelated species (mimicry hypothesis). Based on the investigation we try to estimate which factors potentially affect the evolution of aposematic patterns in *Eniclases*. Our study provides the first insight into the evolution of aposematic signaling in New Guinean net-winged beetles and we try to identify topics for further work.

## Results

### Metriorrhynchina and community structure

**Fig. 3:**
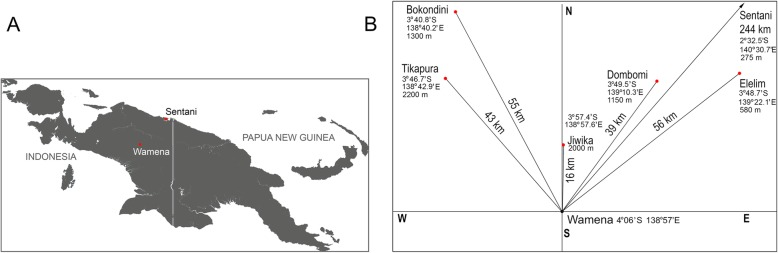
(**a**) Map of New Guinea; (**b**) The relative geographic position of sampled localities, with coordinates and elevation above sea level

**Fig. 4:**
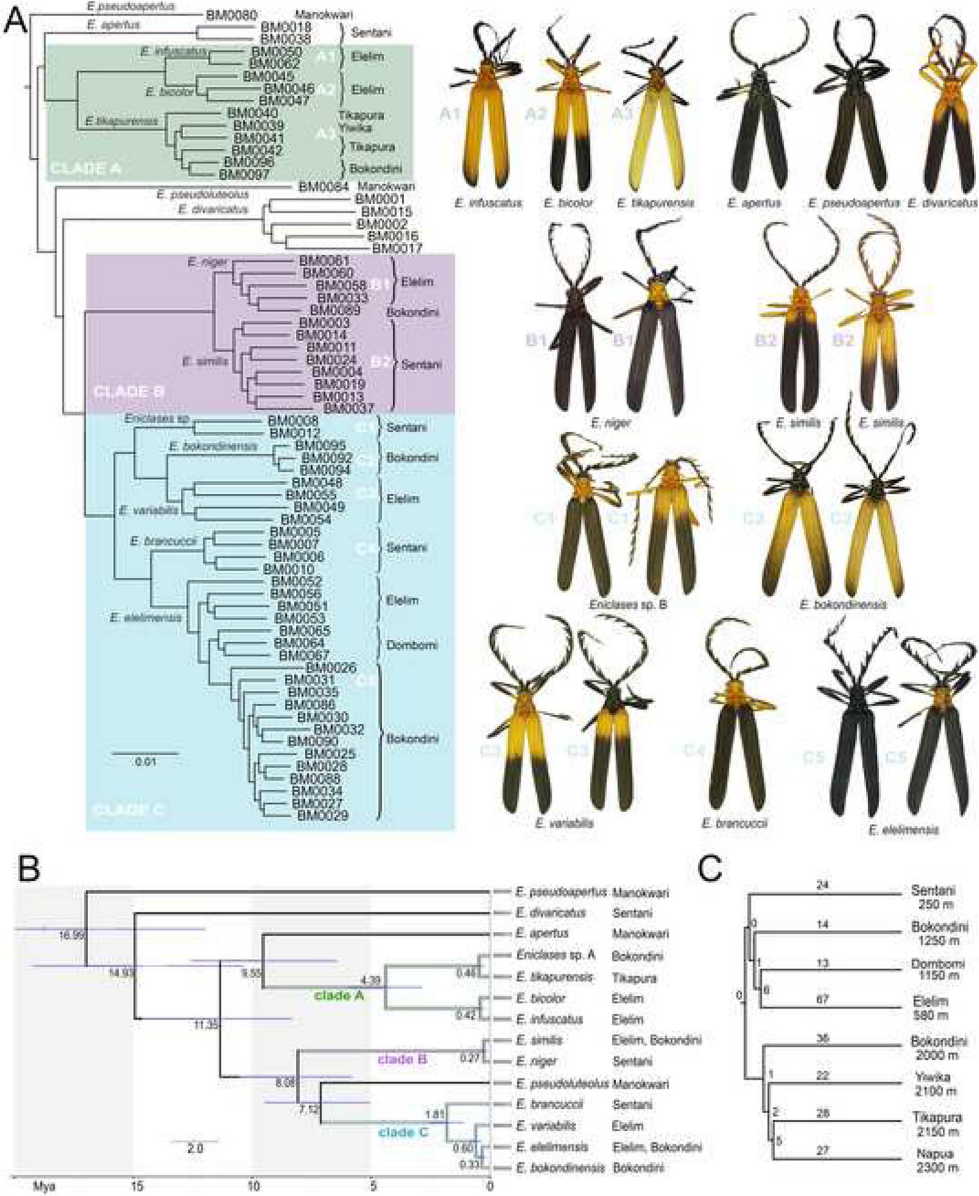
(**a**) – RAD-based maximum likelihood topology with characteristic aposematic patterns for each putative species. The analyzed dataset contained 8.76 × 10^4^ loci and 6.5 × 10^5^ SNPs; all individuals included in the analysis are shown in Additional file 1: Tables S1–S4; (**b**) – Dated phylogenetic tree based on mutation rates of three mitochondrial fragments; (**c**) – Similarity of species composition. The numbers designate share and unique species, respectively

In total, we collected 1914 specimens of Metriorrhynchina beetles from seven localities in New Guinea (Figs. 3a, b). The majority of specimens were collected from aggregations, but some were collected from subsamples from different places (1009 spec., Table 1, Additional file 1: Tables S1, S7). All *Eniclases* and their co-mimics showed limited flying activity and all individuals in aggregations were collected from a few trees in an area < 1000 m^2^. Samples contained 95–433 individuals, 24–91 species total, and up to 6 species of *Eniclases*. Metriorrhynchina is highly diverse in the study area; altogether, 295 species were recorded, 50 trichaline and 12 *Eniclases* species. Most species were recorded from a single locality (231 species, Additional file 1: Table S6), whereas a minority were recorded from two (51 species), three (11 spp.) or four localities (2 spp.). The number of shared species among localities suggested similar species composition within the Wamena valley, i.e., Yiwika, Tikapura, Napua, and Bokondini (2000 m) (Fig. 4c). A group of low- to mid-elevation localities contained Sentani, Bokondini (1250 m), Dombomi, and Elelim. The number of aposematic patterns among all net-winged beetles varied between 3 and 10 at each locality, where only some of these patterns were exhibited by *Eniclases* (Table 1). Non-lycid co-mimics represented < 2% of individuals in each community and belonged to soldier beetles, true bugs, and moths (Fig. 1c, d). They are not discussed further.

**Table 1:** The characteristics of net-winged beetle alpha-diversity and the number of identified color patterns in each locality

Locality, Altitude	AGG	Number of individuals (n)						Number of spp. (n)					# of patterns	
		Total	non-trich.	trichalines %		*Enicl.* %		Total	non- trich.	trich.	*Eniclases* %		Total	*Enicl.*
Sentani 275 m	1	414	77	337	81.4	167	40.3	26	14	12	5	19.2	7	4
Sentani, 360 m	–	19	9	10	52.6	2	10.5	7	3	4	2	22.2	3	2
Elelim, 580 m	2	234	149	85	36.3	20	8.5	85	67	18	6	7.1	10	6
Dombomi (1150)	–	123	89	34	27.6	9	7.3	24	23	1	1	4.2	6	1
Bokondini (1250)	3	110	36	74	67.3	36	32.7	18	9	9	2	11.1	4	2
Bokondini (1287)	4	251	99	152	60.8	43	17.2	13	9	4	2	15.4	3	2
Bokondini (1800)	–	297	171	126	42.4	4	1.3	47	44	3	2	4.3	9	2
Bokondini (2100)	–	24	20	4	16.7	2	8.3	15	14	1	1	6.7	7	1
Tikapura (2170)	–	201	173	28	14.0	7	3.5	53	39	14	1	1.9	8	1
Yiwika (2100)	–	95	78	17	17.9	1	1.1	42	34	8	1	2.4	7	1
Napua (2300)	–	146	138	8	5.5	0	0.0	48	44	4	0	0.0	8	0

n – number of individuals, non-trich. – the genera if Metriorrhynchina except the trichalines; trichaline genera (trichalines) – the genera *Diatrichalus*, *Flabellotrichalus*, *Eniclases*, *Microtrichalus*, *Trichalus*, and *Lobatang*. The numbers for *Eniclases*, are included always also under trichalines as a whole and then separately reported

Trichaline net-winged beetles represented a majority of three aggregations from a lowland foothill forest of the Cyclops mountains (275 m) (81.4%, AGG1, sampling area of ~ 500 m^2^ at a forest margin) and a low mountain forest of the Central Cordillera close to Bokondini (1287 m) (47.3 and 60.8% at AGG3 [300 m^2^] and AGG4 [200 m^2^], respectively). Further communities contained a wider spectrum of Metriorrhynchina and lower representation of trichalines. Samples from a lowland forest close to Elelim (560 m, AGG2) contained 36.3% trichalines, from an area of ~ 1000 m^2^. In total, 763 Metriorrhynchina specimens were collected from higher-elevation mountain forests (Yiwika [2100 m], Bokondini [1700–2100 m], and Tikapura [2150 m]), places known to have a lower abundance of *Eniclases* (Table 1). Species turnover was high among localities, including those which were geographically close (Additional file 1: Tables S6 and S7).

### Presence of color patterns in net-winged beetles communities

All patterns were shared by both sexes and shared patterns were identified in unrelated species (Fig. 4a). Intraspecific variation was identified in 5 of 14 species: *Eniclases niger*, *E. similis*, *E.* sp. B, *E. variabilis*, and *E. elelimensis* (Figs. 4a, Additional file 1: Figures S1–S4). Yellow-black and black patterns were distributed within lowlands and recorded up to ~ 1300 m, whereas pale yellow and pale yellow-dark patterns occurred at > 1500 m. The Dombomi fauna (1150 m) was dominated by a uniform black pattern.

We recorded several discrete mimetic phenotypes in a single place: 3–10 distinct patterns of Metriorrhynchina and 1–6 patterns of *Eniclases* (Table 1; Figs. 1, 2, 4a). Color patterns in *Eniclases* were limited to pale yellow, bright yellow, and orange-yellow bright hues and dark brown to deep black on the upper-body parts. No *Eniclases* were grey, red- or metallic blue-colored, unlike many syntopically occurring Metriorrhynchina (e.g., *Cautiromimus* Fig. 2j, *Carathrix*, *Porrostoma* Fig. 1n, *Diatrichalus* Fig. 1m). Patterns in *Eniclases* closely resembled those of the related genus *Microtrichalus* from the same region (compare *Microtrichalus* sp. in Fig. 2c with *E. apertus*, *E. pseudoapertus*, *E. niger* the right specimen, and *E. elelimensis* the right specimen as they are shown in Fig. 4a. Further, compare *Microtrichalus* sp. in Fig. 2d with *E. bicolor*, *E. similis* the left specimen, and *Eniclases* sp. B the left specimen. Similarly, compare *Microtrichalus* sp. in Fig. 2g with *E. niger* the right specimen, *Eniclases* sp. B the left specimen, and *E. elelimensis* the right specimen. All *Eniclases* are shown in Fig. 4a, at the terminal branches of the phylogram showing their relationships. The transition between bright and dark body parts was sometimes gradual (e.g., *E. infuscatus*, shown in Fig. 4a); in extreme cases, only the apices of elytra were lightly infuscated (*E. tikapurensis*, Fig. 4a), or high contrast was present (*E. variabilis*, the left specimen in Fig. 4a).

### Phylogeny and genetic structure

**Fig. 5:**
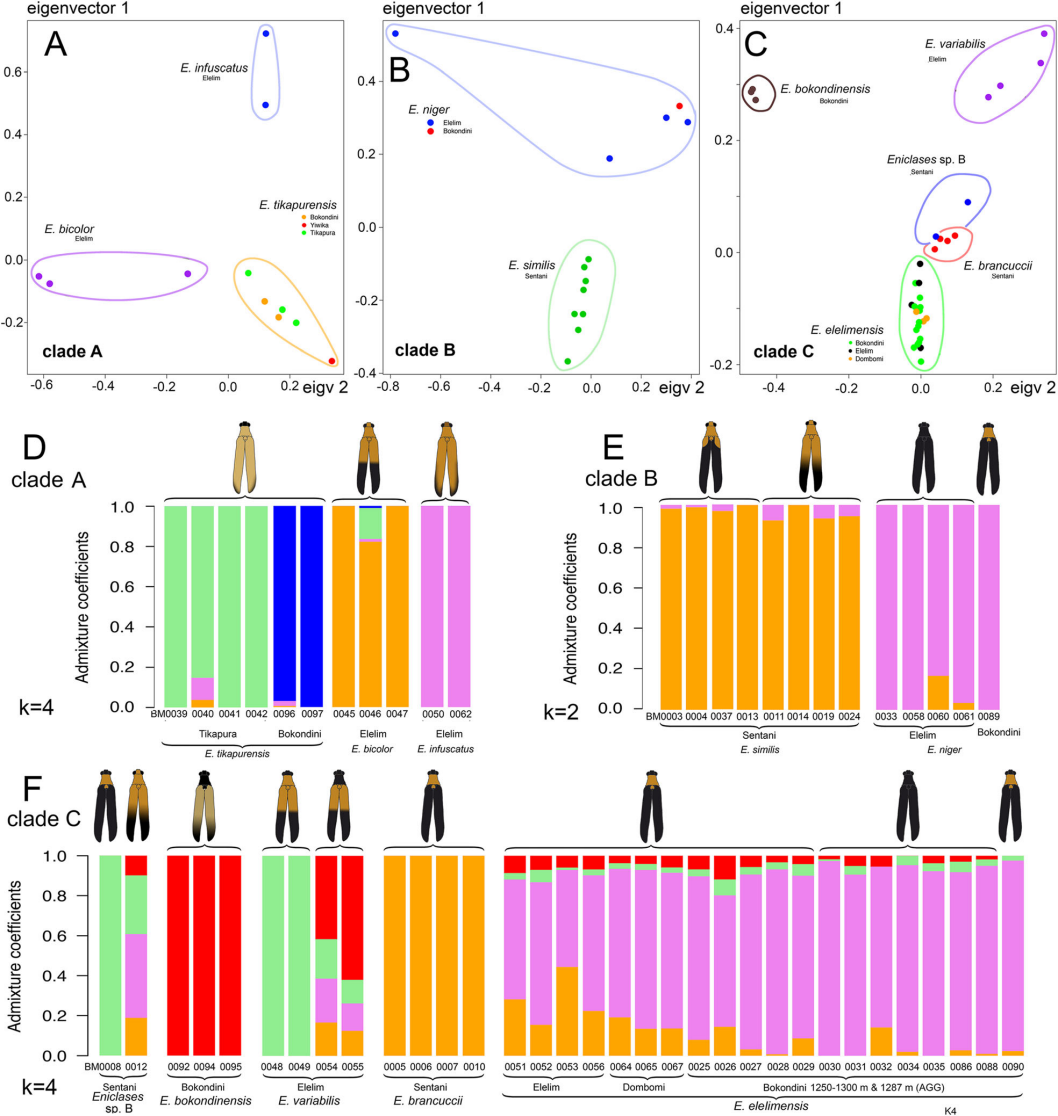
(**a**–**c**) – Distribution of *Eniclases* individuals along principal component (PC) scores of genetic variation based on the analysis of the RAD dataset (PC1 for clades A, B and C 15, 12.1, and 5.6%, respectively; PC2, 15.0, 10.1, and 5.1%, respectively). (**d**–**f**) – Plots of inferred individual’s admixture coefficients based on sparse non-negative matrix factorization (sNMF) implemented in R package LEA for the clades of closely related *Eniclases* species as defined by phylogenetic analyses. Other K clusters are shown in Supplements along with entropy graphs. The analyzed dataset contained 8.76 × 10^4^ loci and 6.5 × 10^5^ SNPs

The nuclear RAD dataset (Wclust = 0.85, MinCov = 4) encompassed 47,000 to 108,000 clusters of possible loci with a mean depth from 5.3 to 32, mainly owing to the low quality of some isolates. As discussed in Methods, we used a Wclust threshold of 0.85 to balance the maximum number of clusters recovered against the loss of individual heterozygosity. The number of clusters generated using a Wclust of 0.85 provided loci with the high heterozygosity level (Additional file 1: Figure S5). The topology produced by the analysis of the Wclust = 0.85 and MinCov = 4 dataset is shown in Fig. 4*A. maximum* likelihood analyses of RAD and mtDNA datasets recovered a highly similar topology (Figs. 4a, b) and the highly similar topology was also recovered when data for individuals belonging to the same clade were separately filtered, a higher number of orthologous loci was identified, and de novo assembled. All analyses supported three clades of closely related species within *Eniclases* further designated as clades A, B, and C (Fig. 4a). Altogether, thirty datasets defined in Table 2 were analyzed using maximum likelihood approach and resulting trees are provided in the newick format in Additional file 2. Deeper splits were poorly supported and are not discussed. Information on numbers of SNPs, positions and loci is summarized in Table 2. Additionally, PCA analyses confirmed the clusters delimited using RAD-based phylogeny (Fig. 5a–c, Additional file 1: Figure S9). Based on the mtDNA mutation rates, the earliest splits within clades A, B, and C were estimated to 4.39, 0.27, and 1.81 mya, respectively (Fig. 4b). Alternative calibration approaches produced only slightly different estimations for critical splits (Additional file 1: Figure S8).

**Table 2:** The characteristics of 66-sample RAD datasets produced under various Wclust and MinCov settings

MinCov number/%		4/7%	8/12%	16/24%	33/50%	48/73%	60/91%
Wclust							
0.70	number of SNPs	407	127.3	24.4	2.56	0.54	0.05
	number of sites	3299	900	164	19.3	4.15	0.44
	number of loci	47.1	12.8	2.29	0.26	0.10	0.01
0.75	number of SNPs	481	157.1	31.7	3.70	1.01	0.16
	number of sites	3906	109.7	205	26.7	7.41	1.12
	number of loci	55.8	15.6	2.87	0.36	0.10	0.02
0.80	number of SNPs	565	194	40.7	4.97	1.45	0.25
	number of sites	4748	1372	259	34.6	10.1	1.69
	number of loci	67.8	19.54	3.64	0.48	0.14	0.02
0.85	number of SNPs	650	240	54.6	6.27	2.09	0.55
	number of sites	6066	1813	356	44.8	15.3	3.79
	number of loci	86.7	25.9	5.0	0.63	0.23	0.05
0.90	number of SNPs	622	241	59.3	7.57	2.60	0.69
	number of sites	7548	2241	444	56.8	20.6	5.31
	number of loci	108.0	32.1	6.33	0.80	0.29	0.08

The numbers of SNPs, sites and loci are given in thousands

## Discussion

Analyses of all data recovered the monophyly of the three clades relevant to this analysis, each consisting of two to five closely related *Eniclases* species (Figs. 4, 5). We identified the recent origin of most *Eniclases* species and mtDNA introgression, indicated by individuals with different nuclear but identical or highly similar mtDNA sequences (Figs. 4, 5). Our species delimitations are based on RAD analyses, as this extensive, genome-wide data can better resolve clusters of individuals with shared ancestry, particularly among closely related species and recently separated populations [30]. Results from sNMF and PCA analyses identified some genomes even with the different genetic structure within the RAD phylogeny-based species-rank entities (Additional file 1: Figures S10–S14) and collectively, the analyses suggested ongoing gene flow and that most species represent recently split lineages, typically within the last million years (Fig. 4b).

### Mimetic patterns in Eniclases

The foundational premise of Müllerian mimicry holds that unprofitable prey benefit from convergence in warning signals [2]. The phylogeny of *Eniclases* showed that resemblance among various *Eniclases* is the product of parallel evolution rather than common ancestry (Fig. 4a). Our data support the Müllerian mimicry hypothesis by the noted sympatric occurrence of distantly related but similarly colored Metriorrhynchina with *Eniclases* (Figs. 1g, h–l, 2). Additionally, the detailed delimitation of species confirms the presence of intraspecific colour polymorphism in these Müllerian mimics with intraspecific forms resembling unrelated *Eniclases* and *Microtrichalus* species.

Aggregations of net-winged beetles in the field indicate that multiple species truly coexist in a single community and do not have a microhabitat-based mosaic distribution. Up to six *Eniclases* aposematic patterns were recorded within single localities in our small study area (Table 1; Fig. 3b) and additional patterns were displayed by closely related Metriorrhynchina (altogether up to 10 patterns in a single locality; Table 1). Unlike findings from other Müllerian mimetic systems [31], these observations suggest that a dominance effect on mimetic polymorphism stemming from microhabitat preference is improbable in *Eniclases*.

Coloration of *Eniclases* is limited to pale to bright yellow, yellowish-orange, and black, but these beetles display multiple distinct color forms with a putative signaling function (Figs. 4a, Additional file 1: Figures S1–S4). Dominant color patterns combine a bright-colored pronotum and elytral humeri with apically dark elytra. Higher contrast is represented by a steeper transition between bright and black parts of the elytra or pronotum. Three species in the study area are black, two of which are polymorphic with some individuals having a yellow pronotum (Fig. 4a). These patterns dominate in all communities containing high representation of trichaline net-winged beetles, generally those < 1500 m in elevation. Additional patterns among high-elevation species are uncommon and include pale yellow or creamy white in combination with a black pronotum and elytral apex (Fig. 4a). *Eniclases* are never red, green, or metallic blue (Figs. 1l-o, 2j–l) and never display three colors, unlike numerous sympatric Metriorrhynchina. It is possible that the absence of red pigment in *Eniclases* is genetically constrained. Earlier studies have shown that easily remembered, high-contrast patterns, e.g., a red-black combination, provide higher protection than lower contrast ones (e.g.*,* [32, 33]. Relative to other net-winged beetles in the same communities, we consider color patterns in *Eniclases* as low contrast, and their effectiveness is likely further reduced by relatively high intraspecific variability, given that 5 of 12 species from Central New Guinea are polymorphic [15] (Fig. 4a, Additional file 1: Figures S1–S4).

Aposematic color patterns are controlled by selection [1, 2] and their distinctness depends on the ability of predators to discriminate among them and on the intensity of predation upon intermediate forms [34]. In contrast to the observed high signal variability, we recorded a distinct, fine-tuned warning signal in *E. divaricatus* and its co-mimics (*E. similis*, *Trichalus* sp., and *Cautiromimus* sp.) represented by characteristic bright-colored humeral patches (Fig. 4a). The specificity of brightly colored humeral patches versus whole humeri color might support the potential role of predators [35] but the present study design cannot provide any experimental evidence. Nevertheless, the structure of *Eniclases* communities shows that under certain conditions, both polymorphism and high pattern similarity can persist within a multi-pattern Müllerian community.

### Behavior and signal perception

Given that trichalines commonly occur in multi-species and multi-pattern aggregations (Table 1), predators encounter a spectrum of aposematic signals related to components such as body shape and colour [12, 36–38]. Further complexity is added to aposematic signaling by different perception of the signal in space and time.

A body shape and size signal are predominantly perceived when an individual sits on the underside of a translucent leaf (Fig. 1a–h). This perception depends on light intensity, microhabitat conditions, and season [15]. We observed that *Eniclases* prefer the undersides of leaves, as do other trichaline genera. We assume that convergent evolution has led to the observed morphological uniformity in body shape and size of > 100 species of trichalines in New Guinea (Additional file 1: Figures S1–S4, Table S4).

Color-based component of signals include hue, uniform or bicolored upper bodies, color patch shape, and the level of contrast between body parts (Figs. 1, 2, 4a). Color is best detected under diffuse-light conditions under a dense forest canopy or when an individual is on upper leaf surfaces, but *Eniclases* and its co-mimics usually avoid upper leaf surfaces, unlike brightly colored lycids such as *Porrostoma*, *Metriorrhynchus*, and *Cladophorus* ([24], field observation). When color pattern is not readily observable, we can assume that the shape component of the signal is relatively stronger. Therefore, the further research is needed to clarify if selection for a shared color pattern might be relaxed. Penny et al. [8] showed the importance of relaxed selection in the origin of imperfect mimicry; we suggest that it may also increase the persistence of a greater number of color patterns in a community (Fig. 4a, Table 1).

Overall, we documented apparent convergence and/or advergence to shared color patterns in distantly related species within *Eniclases* and in unrelated *Microtrichalus* (Figs.2, 4a; compare phenotypically similar and phylogenetically unrelated individuals as described above). Such similarity is usually caused by selection [1, 2, 4–6], which was not studied in this project by experiments. Therefore, excluding similarity due to the common origin, we can speculate that observed similarity of these unpalatable and aposematically coloured beetles is a result of natural selection. In contrast with the Müllerian model which predicts strong selection against rare phenotypes and poor mimics [1–4], we identified multiple examples of intraspecific polymorphism and a high number of different aposematic patterns in a single community. Further detailed research should be focused on the role of factors that can relax or slow the effects of purifying selection, e.g., the generalization of multiple patterns by predators, evolutionary constraints such as the ability, or inability, to rapidly adopt the dominant local aposematic pattern [3, 7, 16, 39] and here suggested behavioral adaptation and variable perception of the body shape and colour (compare Fig. 1a, c, e, g with b, d, f, h showing perception of insect individuals against clear sky and the same individuals illuminated by an artificial light source).

### Eniclases communities in space and time

The model of Müllerian mimicry is simple and earlier experiments have not considered fluctuating conditions during the process of convergence or advergence. Based on our observations, we must assume that the communities in which *Eniclases* occur dynamically change in space due to local conditions and across the altitudinal gradient (Table 1). As a result migrating *Eniclases* commonly enter different mimetic complexes. While we found that similar communities can be expected at similar altitudes in New Guinea, we observed a distinct community composition in the Dombomi locality, which does not contain brightly colored trichalines despite being located at a low elevation. Dombomi is situated on the windward slope of a high mountain range and differs from Elelim in having high levels of precipitation and fog, which supports dense, marshy, medium-height forests with a different fauna. It means that communities with different species and pattern composition might not necessarily be geographically distant.

Additionally, we identified substantial altitude dependent differences in species composition. Despite a distance of less than 2 km, Bokondini communities sampled from 1250 and 1850 m in elevation shared no common species (richness of 26 and 58 species, respectively). Although net-winged beetles are poor dispersers, even low levels of continuous migration could increase the number of aposematic patterns in a community, delay pattern convergence, and increase the number of color-polymorphic species simultaneously adverging to different models [16]. Further research should consider that the communities we analyze today may be products of very different histories, particularly concerning migration.

## Conclusion

*Eniclases* species are uniform in the body size and shape and unrelated species share similar color patterns. In accordance with the explanation of the phenetic uniformity of other unpalatable aposematically coloured insects, we hypothesize that observed similarity of various *Eniclases* was produced by natural selection [1–4], not by common ancestry. Therefore, we considered the diversity of aposematic signals, polymorphism, and coexistence of various patterns within single communities or aggregations of unprofitable net-winged beetles. We suggest that multiple color patterns in net-winged beetles may persist in single communities (1) due to the presence of species that are not able to readily adopt the dominant color pattern (i.e., we observed the absence of red pigment and metallic colours in over 200 species of *Eniclases*, and closely related *Microtrichalus*, *Trichalus*, *Lobatang*, and *Schizotrichalus* [16]) and (2) due to limited perception of colour patterns by predators under some conditions (Figs. 1a–h). Further, we suggest the likelihood of limited, but continuous migration of individuals among populations with different species compositions and pattern frequencies. This hypothesis is based on observed high species turnover between geographically close communities and the presence of a large number of species in communities. As a result of such migration, the number of patterns in the local community would remain continually high and populations or species displaying low-contrast patterns and intraspecific polymorphism could persist for a longer time than in monomorphic Müllerian communities [40]. As we did not perform any experiments under controlled conditions, we cannot conclude if hypothesized selection against rare phenotypes is merely relaxed [41] or is inherently unable to exclude differently colored individuals from complex communities such as those described here. Müllerian mimicry within net-winged beetles is rarely studied, and here we show the potential of Lycidae as an ecologically interesting and highly diverse mimetic lineage.

## Methods

### Field sampling and Eniclases specimens

We collected specimens of 12 *Eniclases* species from seven localities within a small study area (~ 1300 km^2^) in central New Guinea in localities at the northern coast, across an elevation range from sea level to 2170 m (Additional file 1: Tables S1, S3). The majority of individuals were collected in aggregations, designated AGG1-Sentani (275 m), AGG2-Elelim (560 m), AGG3-Bokondini1 (1287 m), and AGG4-Bokondini2 (1250–1300 m). Additional specimens were collected individually in areas with low net-winged beetle abundance. Coloration of the pronotum and elytra were recorded, and patterns were grouped into eight discrete categories (Table 1). All photographs of specimens are provided in Additional file 1: Figures S1–S4.

### Colour pattern classification

Color patterns of New Guinean *Eniclases* were classified into eight categories: (a) uniform black; (b) pronotum orange, elytra black, (c) pronotum and humeral patches orange, remainder of elytra black; (d) pronotum and humeral part of elytra orange, remainder of elytra black; (e) pronotum black, humeral part of elytra orange, remainder of elytra black; (f) pronotum black, the humeral part of elytra light yellow, apex infuscate; (g) pronotum and elytra orange to light yellow, the apex of elytra infuscate, and; (h) uniform yellow.

### Laboratory procedures

Total DNA was extracted using a Wizard SV96 Purification System (Promega Inc.). Extraction yields were measured using a NanoDrop-1000 Spectrophotometer. The fragments *rrnL* + *tRNA-Leu* + *nad1* (~ 830 bp) and *nad5* + tRNAs (~ 1210 bp) mitochondrial DNA (mtDNA) were amplified. Primers are listed in Additional file 1: Table S2 and polymerase chain reaction (PCR) settings followed Sklenarova et al. [21]. PCR products were purified using PCRμ96TM Plates (Millipore Inc.) and sequenced by an ABI3130 automated sequencer using a BigDye® Terminator Cycle Sequencing Kit 1.1. Sequences were deposited in the GenBank database (accession numbers KT265092–KT265172, MF288197–MF288482 and MG844591–MF844955).

### MtDNA data sampling and phylogenetic analyses

Earlier published sequences of *cox1* mtDNA [42] were merged with *rrnL* and *nad5* fragments. Fragments were aligned separately using MAFFT v. 7.017 [43] in Geneious 7.1.9 (Biomatters, Ltd., Auckland, New Zealand) and the concatenated dataset was analyzed to infer a phylogenetic tree. We used IQ-TREE v. 1.6.6 [44] to estimate mtDNA phylogeny using an ultrafast bootstrap approximation and 5000 iterations. The best models for each fragment were selected in IQ-TREE using ModelFinder [45] (Additional file 1: Table S3).

A tree pruned to one representative per species was dated using a Bayesian approach implemented in BEAST 1.8.1 [46]. We produced 5 × 10^7^ generations with sampling every 2500 generations. Only the *rrnL*, *cox1*, and *nad5* genes were analyzed and genes and codon positions were partitioned (Additional file 1: Table S3). Each partition was provided with its own parameters. Due to an absent fossil record and young relevant splits, i.e., younger than five million years, we used mtDNA rate information to calibrate our topology: 0.0115 subs/s/my/l for *cox1*, 0.0177 subs/s/my/l for *nad5*, and 0.0054 subs/s/my/l for *rrnL* [47]. To estimate the possible effects of a higher rate of mutations, we repeated the analyses of the *cox*1 fragment with a doubled rate (0.023 subs/s/my/l). The best topology recovered from maximum likelihood analyses was fixed by a guiding tree and switching off tree operators during analyses. Convergence was assessed in Tracer v. 1.7 [48] and the first 1.25 × 10^7^ generations were set as a burn-in.

### Next-RAD sampling and analyses

Based on the mtDNA analyses, we selected 66 individuals for subsequent RAD sequencing. The samples represent 14 species of *Eniclases* net-winged beetles from seven sites. We used a high number of genomic loci across closely related species and populations as they could provide resolution on recent species-level interactions and support the phylogenetic hypothesis [49, 50]. Given that a reference genome is unavailable for RAD data masking due to its size (3–5 GB), de novo assembly with clustering thresholds (Wclust, degree of sequence similarity) was used to search for orthologous sequences [51]. The nextRAD genomic sequencing was provided by SNPsaurus Inc., where the Illumina Hi-Seq system was used to generate data. The DNA next to a restriction site GTGTAGAGG was sequenced. RAD sequencing produces individually barcoded single-end read amplifications with an average length of ~ 75 bp representing loci scattered across the genome. Each read was individually assigned to the specific specimen voucher. Illumina reads were deposited in the Sequence Read Archive (PRJNA544184).

Quality of raw Illumina reads were visualized by FastQC. We used the software iPYRAD 0.6.24 [52] to de-multiplex, trim, filter and identify a de novo assembly of orthologous loci. This software uses an alignment-clustering method involving indel variation, which improves the precision of recognition of global homology across different samples, and read trimming, which generates variable read lengths unlike alternative assembly methods, e.g., Stacks [53, 54]. First, all Illumina adapters were removed from data. The data were processed for all individuals with separate identifiers and the reads without identifier were excluded. Then, bases were trimmed from the 3′ end of reads when the quality score was below 30. The minimum depth for the base call was set to 6. Other parameters were set to default values.

The maximum size of the data matrix varied as we varied the Wclust parameter. We tested these matrices by analyzing five Wclust settings from 0.7–0.9, increasing by 0.05 for each filtering. The number of potential loci increased when Wclust increased, although within-individual heterozygosity decreased significantly when Wclust was set higher than 0.85 (Additional file 1: Figure S5). To balance the highest proportion of potential loci accepted and the highest rate of sample heterozygosity, a Wclust value of 0.85 was used in the final analyses. A minimum depth (MinDepth) of six reads, together with a minimum number of four samples that contained data for a given locus (minimum taxon coverage, MinCov) was used in the final dataset whose analysis was used for discussion. The proportion of missing data and the number of loci filtered may affect the recovered topology. These characteristics are strongly dependent on the MinCov parameter [55], therefore, we also produced additional data matrices with varied MinCov and Wclust values. Altogether, 30 datasets with unique settings were generated (Wclust from 0.7–0.9 increasing by 0.05 and MinCov of 4, 8, 16, 33, 48 and 60 for each unique filtering). To test the congruence of topologies within individual clades of closely related species, data were filtered independently for clades A through C, as defined by preliminary analyses. We inferred individual admixture coefficients based on sparse non-negative matrix factorization (sNMF) analyses using the package ‘LEA’ in R [56] on a dataset with a Wclust of 0.85 and a MinCov of 4 to reveal population genetic structure. We evaluated cross-entropy criterion for clusters K = 1–10 using the obj.snmf function in LEA [56]. We further performed principal components analysis (PCA) in R, using the package ‘SNPRelate’ 1.6.4 [57] to visualize the major axes of genetic variation using the above dataset, reduced by linkage-disequilibrium-based single nucleotide polymorphism (SNP) pruning as implemented in the package ‘SNPRelate’, the command snpgdsLDpruning and ld.threshold = 0.1.

Each matrix generated with a specific Wclust and MinCov values was used to infer a maximum likelihood phylogenetic tree using same settings as described above. We analyzed them with a maximum likelihood approach using IQ-TREE, with an ultrafast bootstrap approximation and 5000 iterations. ModelFinder, implemented in IQ-TREE, estimated the optional evolution model for final matrix (Additional file 1: Table S3). Resulting tree topologies from all data matrices were examined and are provided in the Additional file 2 “The resulting tree topologies from all data matrices recovered from RAD data filtering”. In addition to maximum likelihood trees inferred using IQ-TREE, we used SVDquartets [58], implemented in PAUP* (v. 4.0a, build 165 [59];), and evaluated bootstrap support over 1000 iterations. In the final step, the PAUP* version of the QFM algorithm [60] was used to search for the overall tree that minimized the number of quartets that were inconsistent with it. All produced topologies were checked to identify possible incongruences.

## Additional files

Additional file 1: **Table S1.** The list of samples with information on geographic origins and color patterns. **Table S2.** Primers used for mtDNA amplification. **Table S3.** Characteristics of datasets and best-fit models for mtDNA and nextRAD partitions. **Table S4** Measurements of Eniclases. **Table S5.** Euclidian distances among sampled localities in central New Guinea. **Table S6.** The species structure of Metriorrhynchina communities. **Figures S1–S4.** Aposematic patterns of sequenced specimens from northern New Guinea (parts 1–4). **Figure S5.** Testing of the effect of clustering threshold on individual heterozygosity and proportion of loci generated. **Figure S6.** The full resolution RAD-based tree. **Figure S7.** The full resolution RAD-based analyses of individual subclades based on subset read filtering. **Figure S8.** The dated tree based on mtDNA dataset. **Figure S9.** Principal component analysis of three defined clades A, B and C based on RAD dataset. **Figures S10–S14.** Plots of individual’s admixture coefficients based on sparse non-negative matrix factorization (sNMF) implemented in R package LEA for the specific clades.

Additional file 2: The resulting tree topologies from all data matrices recovered from RAD data filtering. The trees are provided in the newick format.

## Data Availability

Sequence data can be found NCBI Sequence Read Archive (SRA; ABCDE111111) and GenBank (mtDNA data, KT265092–KT265172, MF288197–MF288482, MG844591–MF844955).

## Abbreviations

AGG: Aggregation
DNA: Deoxyribonucleic acid
MinCov: Minimal coverage
MinDepth: Minimum depth
PCA: Principal component analysis
PCR: Polymerase chain reaction
RAD: Restriction site associated DNA markers
sNMF: sparse non-negative matrix factorization
Wclust: clustering threshold as a decimal number
