# Supplementary material for: Persistence of multiple patterns and intraspecific polymorphism in multi-species Müllerian communities of net-winged beetles
Source: Front Zool. 2019 Oct 17;16:38. doi: 10.1186/s12983-019-0335-8 (PMC6798367; doi:10.1186/s12983-019-0335-8)
Supplement: Supplementary file 1 — Table S1. The list of samples with information on geographic origins and color patterns. Table S2. Primers used for mtDNA amplification. Table S3. Characteristics of datasets and best-fit models for mtDNA and nextRAD partitions. Table S4 Measurements of Eniclases. Table S5. Euclidian distances among sampled localities in central New Guinea. Table S6. The species structure of Metriorrhynchina communities. Figures S1–S4. Aposematic patterns of sequenced specimens from northern New Guinea (parts 1–4). Figure S5. Testing of the effect of clustering threshold on individual heterozygosity and proportion of loci generated. Figure S6. The full resolution RAD-based tree. Figure S7. The full resolution RAD-based analyses of individual subclades based on subset read filtering. Figure S8. The dated tree based on mtDNA dataset. Figure S9. Principal component analysis of three defined clades A, B and C based on RAD dataset. Figures S10–S14. Plots of individual’s admixture coefficients based on sparse non-negative matrix factorization (sNMF) implemented in R package LEA for the specific clades. [file 12983_2019_335_MOESM1_ESM.pdf]

Persistence of multiple patterns and intraspecific polymorphism in net-winged beetles due to variable signal perceptions and community structures

Matej Bocek, Dominik Kusy, Michal Motyka & Ladislav Bocak

The list of supplementary material.

Supplementary Tables

Table S1. The list of samples with information on geographic origins and color patterns.
Table S2. Primers used for mtDNA amplification.
Table S3. Characteristics of datasets and best-fit models for mtDNA and nextRAD partitions.
Table S4. Measurements of *Eniclases*.
Table S5. Euclidian distances among sampled localities in central New Guinea.
Table S6. The species structure of Metriorrhynchina communities.

Supplementary Figures

Figure S1. Aposematic patterns of sequenced specimens from northern New Guinea (part 1)
Figure S2. Aposematic patterns of sequenced specimens from northern New Guinea (part 2)
Figure S3. Aposematic patterns of sequenced specimens from northern New Guinea (part 3)
Figure S4. Aposematic patterns of sequenced specimens from northern New Guinea (part 4)
Figure S5. Testing of the effect of clustering threshold on individual heterozygosity and proportion of loci generated.
Figure S6. The full resolution RAD-based tree.
Figure S7. The full resolution RAD-based analyses of individual subclades based on subset read filtering.
Figure S8. The dated tree based on mtDNA dataset.
Figure S9. Principal component analysis of three defined clades A, B and C based on RAD dataset.
Figure S10–S14. Plots of individual's admixture coefficients based on sparse non-negative matrix factorization (sNMF) implemented in R package LEA for the specific clades.

Supplementary Information The resulting tree topologies from all data matrices recovered from RAD data filtering. The trees are provided in the newick format.

Table S1. The list of species, their geographic origins, collecting circumstances and aposematic patterns.

✹ Individuals with incongruent species placement in mtDNA and RAD analyses P Individuals belonging to a polymorphic species based on the RAD phylogeny

Eniclases species identification<br>Genomic		 mtDNA and morph.	Voucher<br>number	Locality<br>(all New Guinea, Papua Province if not stated otherwise)	Aggregation	Colour<br>pattern
E. pseudoapertus		*E. pseudoapertus*	BM0080	West Papua, Manokwari distr., Maibri vill., Arfak Mts., 1570 m	-	Fig. 7
E. pseudoluteolus		*E. pseudoluteolus*	BM0084	West Papua, Manokwari distr., Maibri vill., Arfak Mts., 1570 m	-	uniform yellow
E. divaricatus		*E. divaricatus*	BM0057	Elelim, km 5 rd to Apalapsili, 03°48.700´S, 139°22.088´E, 580 m		similar to Fig. 6
E. divaricatus		*E. divaricatus*	BM0001	Sentani, S slope of Cyclop Mts., 02°32.487´S, 140°30.683´E, 275 m	AGG1	similar to Fig. 6
E. divaricatus		*E. divaricatus*	BM0009	Sentani, S slope of Cyclop Mts., 02°32.487´S, 140°30.683´E, 275 m	AGG1	similar to Fig. 6
E. divaricatus		*E. divaricatus*	BM0015	Sentani, S slope of Cyclop Mts., 02°32.487´S, 140°30.683´E, 275 m	AGG1	similar to Fig. 6
E. divaricatus		*E. divaricatus*	BM0017	Sentani, S slope of Cyclop Mts., 02°32.487´S, 140°30.683´E, 275 m	AGG1	similar to Fig. 6
E. divaricatus		*E. divaricatus*	BM0016	Sentani, S slope of Cyclop Mts., 02°32.487´S, 140°30.683´E, 275 m	AGG1	Fig. 6
E. divaricatus		*E. divaricatus*	BM0002	Sentani, S slope of Cyclop Mts., 02°32.487´S, 140°30.683´E, 275 m	AGG1	similar to Fig. 6
E. apertus		*E. apertus*	BM0038	Sentani, S slope Cyclop Mts., 02°32.320´S, 140°30.738´E, 360 m	-	Fig. 8
E. apertus		*E. apertus*	BM0018	Sentani, S slope of Cyclop Mts., 02°32.487´S, 140°30.683´E, 275 m	AGG1	similar to Fig. 8
**Clade A**						
E. niger	P	*E. niger*	BM0089	Bokondini (AGG), 03°40.76´S, 138°40.15´E, 1287 m	AGG3	Fig. 15
E. niger	P	*E. niger*	BM0087	Bokondini (AGG), 03°40.76´S, 138°40.15´E, 1287 m	AGG3	intermediate, as Figs 14 and 15
E. niger	P	*E. niger*	BM0060	Elelim, km 5 rd to Apalapsili, 03°48.700´S, 139°22.088´E, 580 m	AGG2	similar to Fig. 14
E. niger	P	*E. niger*	BM0059	Elelim, km 5 rd to Apalapsili, 03°48.700´S, 139°22.088´E, 580 m	AGG2	Fig. 14
E. niger	P	*E. niger*	BM0061	Elelim, km 5 rd to Apalapsili, 03°48.700´S, 139°22.088´E, 580 m	AGG2	similar to Fig. 14
E. niger	P	*E. niger*	BM0033	Bokondini, 03°40.76´S, 138°40.15´E, 1250-1300 m	AGG4	similar to Fig. 14
E. niger	P	*E. niger*	BM0058	Elelim, km 5 rd to Apalapsili, 03°48.700´S, 139°22.088´E, 580 m	AGG2	similar to Fig. 14
E. similis	P	*E. similis*	BM0024	Sentani, S slope of Cyclop Mts., 02°32.487´S, 140°30.683´E, 275 m	AGG1	Fig. 13
E. similis	P	*E. similis*	BM0037	Sentani, S slope Cyclop Mts., 02°32.320´S, 140°30.738´E, 360 m	-	similar to Fig. 13
E. similis	P	*E. similis*	BM0020	Sentani, S slope of Cyclop Mts., 02°32.487´S, 140°30.683´E, 275 m	AGG1	intermediate, as Figs 12 and 13
E. similis	P	*E. similis*	BM0019	Sentani, S slope of Cyclop Mts., 02°32.487´S, 140°30.683´E, 275 m	AGG1	intermediate, as Figs 12 and 13
E. similis	P	*E. similis*	BM0022	Sentani, S slope of Cyclop Mts., 02°32.487´S, 140°30.683´E, 275 m	AGG1	intermediate, as Figs 12 and 13
E. similis	P	*E. similis*	BM0021	Sentani, S slope of Cyclop Mts., 02°32.487´S, 140°30.683´E, 275 m	AGG1	similar to Fig. 12
E. similis	P	*E. similis*	BM0023	Sentani, S slope of Cyclop Mts., 02°32.487´S, 140°30.683´E, 275 m	AGG1	intermediate, as Figs 12 and 13
E. similis	P	*E. similis*	BM0003	Sentani, S slope of Cyclop Mts., 02°32.487´S, 140°30.683´E, 275 m	AGG1	Fig. 12
E. similis	P	*E. similis*	BM0014	Sentani, S slope of Cyclop Mts., 02°32.487´S, 140°30.683´E, 275 m	AGG1	intermediate, as Figs 12 and 13
E. similis	P	*E. similis*	BM0013	Sentani, S slope of Cyclop Mts., 02°32.487´S, 140°30.683´E, 275 m	AGG1	similar to Fig. 12
E. similis	P	*E. similis*	BM0011	Sentani, S slope of Cyclop Mts., 02°32.487´S, 140°30.683´E, 275 m	AGG1	similar to Fig. 13
E. similis	P	*E. similis*	BM0004	Sentani, S slope of Cyclop Mts., 02°32.487´S, 140°30.683´E, 275 m	AGG1	similar to Fig. 12

**Clade B**					
E. infuscatus	*E. infuscatus*	BM0050	Elelim, km 5 rd to Apalapsili, 03°48.700´S, 139°22.088´E, 580 m	AGG2	Fig. 11
E. infuscatus	*E. infuscatus*	BM0062	Elelim, km 6 rd to Apalapsili, 03°48.686´S, 139°21.764´E, 650 m	-	similar to Fig. 11
E. bicolor	*E. bicolor*	BM0045	Elelim, km 5 rd to Apalapsili, 03°48.700´S, 139°22.088´E, 580 m	AGG2	similar to Fig. 10
E. bicolor	*E. bicolor*	BM0046	Elelim, km 5 rd to Apalapsili, 03°48.700´S, 139°22.088´E, 580 m	AGG2	Fig. 10
E. bicolor	*E. bicolor*	BM0047	Elelim, km 5 rd to Apalapsili, 03°48.700´S, 139°22.088´E, 580 m	AGG2	similar to Fig. 10
E. tikapurensis	✹*Eniclases* sp. A	BM0093	3 km N Bokondini, 03°39.741´S, 138°40.216´E, 1750–1900 m	-	similar to Fig. 9
E. tikapurensis	✹*Eniclases* sp. A	BM0097	3 km SW Bokondini, 03°42.51´S, 138°38.893´E, 2100 m	-	similar to Fig. 9
E. tikapurensis	✹*Eniclases* sp. A	BM0096	3 km SW Bokondini, 03°42.51´S, 138°38.893´E, 2100 m	-	similar to Fig. 9
E. tikapurensis	*E. tikapurensis*	BM0039	Yiwika, 16 km N Wamena, 03°56.883´S, 138°57.712´E, 2100 m	-	Fig. 9
E. tikapurensis	*E. tikapurensis*	BM0043	Tikapura (Rd Tagime-Kelila), 03°46.797'S, 138°42.933´E, 2170 m	-	similar to Fig. 9
E. tikapurensis	*E. tikapurensis*	BM0040	Tikapura (Rd Tagime-Kelila), 03°46.797'S, 138°42.933´E, 2170 m	-	similar to Fig. 9
E. tikapurensis	*E. tikapurensis*	BM0044	Tikapura (Rd Tagime-Kelila), 03°46.797'S, 138°42.933´E, 2170 m	-	similar to Fig. 9
E. tikapurensis	*E. tikapurensis*	BM0042	Tikapura (Rd Tagime-Kelila), 03°46.797'S, 138°42.933´E, 2170 m	-	similar to Fig. 9
E. tikapurensis	*E. tikapurensis*	BM0041	Tikapura (Rd Tagime-Kelila), 03°46.797'S, 138°42.933´E, 2170 m	-	similar to Fig. 9

**Clade C**					
Eniclases sp. B P	✹*E. variabilis*	BM0012	Sentani, S slope of Cyclop Mts., 02°32.487´S, 140°30.683´E, 275 m	AGG1	Fig. 26
Eniclases sp. B P	✹*E. variabilis*	BM0008	Sentani, S slope of Cyclop Mts., 02°32.487´S, 140°30.683´E, 275 m	AGG1	Fig. 25
E.brancuccii	*E.brancuccii*	BM0006	Sentani, S slope of Cyclop Mts., 02°32.487´S, 140°30.683´E, 275 m	AGG1	similar to Fig. 16
E.brancuccii	*E.brancuccii*	BM0005	Sentani, S slope of Cyclop Mts., 02°32.487´S, 140°30.683´E, 275 m	AGG1	Fig. 16
E.brancuccii	*E.brancuccii*	BM0010	Sentani, S slope of Cyclop Mts., 02°32.487´S, 140°30.683´E, 275 m	AGG1	similar to Fig. 16
E.brancuccii	*E.brancuccii*	BM0007	Sentani, S slope of Cyclop Mts., 02°32.487´S, 140°30.683´E, 275 m	AGG1	similar to Fig. 16
E. bokondinensis	*E. bokondinensis*	BM0092	3 km N Bokondini, 03°39.741´S, 138°40.216´E, 1750–1900 m	-	Fig. 18
E. bokondinensis	*E. bokondinensis*	BM0094	3 km N Bokondini, 03°39.741´S, 138°40.216´E, 1750–1900 m	-	similar to Fig. 17
E. bokondinensis	*E. bokondinensis*	BM0095	3 km N Bokondini, 03°39.741´S, 138°40.216´E, 1750–1900 m	-	Fig. 17
E. variabilis P	*E. variabilis*	BM0054	Elelim, km 5 rd to Apalapsili, 03°48.700´S, 139°22.088´E, 580 m	AGG2	Fig. 20
E. variabilis P	*E. variabilis*	BM0055	Elelim, km 5 rd to Apalapsili, 03°48.700´S, 139°22.088´E, 580 m	AGG2	Fig. 21
E. variabilis P	*E. variabilis*	BM0048	Elelim, km 5 rd to Apalapsili, 03°48.700´S, 139°22.088´E, 580 m	AGG2	Fig. 23
E. variabilis P	*E. variabilis*	BM0049	Elelim, km 5 rd to Apalapsili, 03°48.700´S, 139°22.088´E, 580 m	AGG2	Fig. 22
E. elelimensis P	*E. elelimensis*	BM0052	Elelim, km 5 rd to Apalapsili, 03°48.700´S, 139°22.088´E, 580 m	AGG2	similar to Fig. 19
E. elelimensis P	*E. elelimensis*	BM0051	Elelim, km 5 rd to Apalapsili, 03°48.700´S, 139°22.088´E, 580 m	AGG2	similar to Fig. 19
E. elelimensis P	*E. elelimensis*	BM0056	Elelim, km 5 rd to Apalapsili, 03°48.700´S, 139°22.088´E, 580 m	AGG2	Fig. 19
E. elelimensis P	✹*E. variabilis*	BM0053	Elelim, km 5 rd to Apalapsili, 03°48.700´S, 139°22.088´E, 580 m	AGG2	Fig. 24

E. elelimensis	P	✸*E. variabilis*	BM0035	Bokondini, 03°40.76´S, 138°40.15´E, 1250–1300 m	-	Fig. 27
E. elelimensis	P	✸*E. variabilis*	BM0029	Bokondini, 03°40.76´S, 138°40.15´E, 1250–1300 m	AGG4	similar to Fig. 28
E. elelimensis	P	✸*E. variabilis*	BM0028	Bokondini, 03°40.76´S, 138°40.15´E, 1250–1300 m	AGG4	similar to Fig. 28
E. elelimensis	P	✸*E. variabilis*	BM0027	Bokondini, 03°40.76´S, 138°40.15´E, 1250–1300 m	AGG4	similar to Fig. 28
E. elelimensis	P	✸*E. variabilis*	BM0091	Bokondini (AGG), 03°40.76´S, 138°40.15´E, 1287 m	AGG3	similar to Fig. 25
E. elelimensis	P	✸*E. variabilis*	BM0090	Bokondini (AGG), 03°40.76´S, 138°40.15´E, 1287 m	AGG3	similar to Fig. 25
E. elelimensis	P	✸*E. variabilis*	BM0088	Bokondini (AGG), 03°40.76´S, 138°40.15´E, 1287 m	AGG3	similar to Fig. 27
E. elelimensis	P	✸*E. variabilis*	BM0086	Bokondini (AGG), 03°40.76´S, 138°40.15´E, 1287 m	AGG3	similar to Fig. 27
E. elelimensis	P	✸*E. variabilis*	BM0036	Bokondini, 03°40.76´S, 138°40.15´E, 1250-1300 m	AGG4	similar to Fig. 27
E. elelimensis	P	✸*E. variabilis*	BM0063	Dombomi, Lower Pass vall., 03°49.477´S, 139°10.251´E, 1150 m	-	similar to Fig. 29
E. elelimensis	P	✸*E. variabilis*	BM0065	Dombomi, Lower Pass vall., 03°49.477´S, 139°10.251´E, 1150 m		similar to Fig. 29
E. elelimensis	P	✸*E. variabilis*	BM0066	Dombomi, Lower Pass vall., 03°49.477´S, 139°10.251´E, 1150 m		similar to Fig. 2
E. elelimensis	P	✸*E. variabilis*	BM0067	Dombomi, Lower Pass vall., 03°49.477´S, 139°10.251´E, 1150 m		similar to Fig. 29
E. elelimensis	P	✸*E. variabilis*	BM0064	Dombomi, Lower Pass vall., 03°49.477´S, 139°10.251´E, 1150 m		Fig. 29
E. elelimensis	P	✸*E. variabilis*	BM0026	Bokondini, 03°40.76´S, 138°40.15´E, 1250–1300 m	AGG4	similar to Fig. 28
E. elelimensis	P	✸*E. variabilis*	BM0025	Bokondini, 03°40.76´S, 138°40.15´E, 1250–1300 m	AGG4	Fig. 28
E. elelimensis	P	✸*E. variabilis*	BM0030	Bokondini, 03°40.76´S, 138°40.15´E, 1250–1300 m	AGG4	similar to Fig. 28
E. elelimensis	P	✸*E. variabilis*	BM0031	Bokondini, 03°40.76´S, 138°40.15´E, 1250–1300 m	AGG4	similar to Fig. 27
E. elelimensis	P	✸*E. variabilis*	BM0034	Bokondini, 03°40.76´S, 138°40.15´E, 1250–1300 m	AGG4	similar to Fig. 27
E. elelimensis	P	✸*E. variabilis*	BM0032	Bokondini, 03°40.76´S, 138°40.15´E, 1250–1300 m	AGG4	similar to Fig. 27

Table S2. The list of primers used for mtDNA amplification.

Fragment	Code	-mer	Sequence (5' >> 3')
rrnL	16a	20	CGC CTG TTT AAC AAA AAC AT
	ND1A	27	GGT CCC TTA CGA ATT TGA ATA TAT CCT
	ND1-2	24	ATC AAA AGG AGC TCG ATT AGT TTC
cox1	JerryN	23	CAA CAY YTA TTY TGA TTY TTY GG
	MarcyN	24	TTC RTA WGT TCA RTA TCA TTG RTG
	JerryM	23	CAA CAY YTA TTT TGR TTY TTT GG
	Marcy	27	TAR TTC RTA TGW RCA ATA YCA YTG RTG
	SPat	21	GCA CTA WTC TGC CAT ATT AGA
	SJerry	23	CAA CAT YTA TTY TGA TTY TTT GG
	Pat	25	TCC ATT GCA CTA ATC TGC CAT ATT A
	Jerry	23	CAA CAT TTA TTT TGA TTT TTT
	Marilyn	21	TCA TAA GTT CAG TAT CAT TG
nad5	OF1	29	CCT ACT CCT GTT TCT GCT TTA GTT CAT TC
	R6	29	GAA ACG AAA AAT CGT ATT TAA TTT CGA CT
	R2M	29	AAT TGA ASC CAA AAA GAG GTA TAT CAC TG

Table S3. Characteristics of datasets and best-fit models for mtDNA and nextRAD partitions.

mtDNA dataset analysis

Fragment Name	Number of sequences	Sites	Unique	Informative	Constant
rrnL	63	796	99	83	679
cox1	64	1101	227	249	807
nad5	62	1207	272	299	864

Name	Model	LogL	AIC	w-AIC	AICc	w-AICc	BIC	w-BIC
rrnL	TIM3+F+I	-1983.3195	4226.6390 + 0.0000		4277.8571 + 0.0000		4834.9869 + 0.0000	
cox1	TIM2+F+I+G4	-4175.5849	8617.1699 + 0.0000		8654.0303 + 0.0000		9282.6984 + 0.0000	
nad5	HKY+F+I+G4	-4303.4783	8860.9565 + 0.0000		8891.0881 + 0.0000		9508.1350 + 0.0000	

nextRAD dataset alignment analysis (Wclust = 0.85, MinCov = 4).

Input data: 66 sequences with 6066010 nucleotide sites
Number of constant sites: 5.58659 x 10^6 (= 92.0966 % of all sites)
Number of invariant (constant or ambiguous constant) sites: 5.58659 x 10^6 (= 92.0966 % of all sites)
Number of parsimony informative sites: 154300
Number of distinct site patterns: 942024

Model of substitution: GTR+F+I+G4

Rate parameter R:
A-C: 0.9833
A-G: 3.6524
A-T: 0.9157
C-G: 0.9335
C-T: 3.7609
G-T: 1.0000

	State frequencies	Rate matrix Q:			
A	pi(A) = 0.2936	-0.9256	0.1451	0.6	0.1806
C	pi(C) = 0.2047	0.208	-1.103	0.1533	0.7419
G	pi(G) = 0.2279	0.7727	0.1377	-1.108	0.1973
T	pi(T) = 0.2737	0.1937	0.5548	0.1643	-0.9128

Model of rate heterogeneity: Invar+Gamma with 4 categories
Proportion of invariable sites: 0.6185
Gamma shape alpha: 0.7601

Category	Relative_rate	Proportion
0	0	0.6185
1	0.2282	0.09538
2	1.024	0.09538
3	2.478	0.09538
4	6.755	0.09538

Relative rates are computed as MEAN of the portion of the Gamma distribution falling in the category.

Table S4. Measurements of *Eniclases* spp. (n.a. – not available; (**b**) - uniformly black colored species)

Species included in the current DNA analyses

Species	Body length	Width humeri	Pronotum length	Pronotum width	EDiam/Edist male
E. pseudoapertus(**b**)	6.3	1.6	0.75	1.2	1.4
E. divaricatus	6.8–9.7	2.1–2.3	1.2–1.3	1.7–1.7	0.92–0.96
E. pseudoluteolus	9.3	2.3	1.15	1.6	0.9
E. apertus	5.7–8.4	1.34–1.7	0.9	1.25	1.15–1.17
E. tikapurensis	9.5–11.1	2.0–2.5	1.1–1.3	1.4–1.7	1.11–1.40
E. bicolor	10.3	2.4	1.4	1.7	n.a.
E. infuscatus	12.1	2.5	1.25	1.6	n.a.
E. brancuccii	7.6–8.0	1.8–1.9	1.0–1.1	1.5–1.8	1.00
E. bokondinensis	9.2	2.05	1.0	1.35	n.a.
E. elelimensis	6.9–8.1	1.5–1.9	0.9–1.1	1.3–1.4	0.73–0.86
E. variabilis	6.6–8.2	1.6–2.0	0.1–1.1	1.1–1.35	0.90–0.96
E. niger (**b**)	9.2–11.6	2.2–2.8	1.3–1.6	9.0–11.5	1.17–1.28
E. similis	7.5–9.7	1.9–2.3	1.1–1.4	1.8	1.02–1.15

Other species of *Eniclases* (from Bocak & Bocakova 1991)

E. efferatus	6.8-9.2	1.5–1.9			1.00
E. egregius	8.5–9.2	2.2–2.4			1.08
E. electus	6.7–8.0	1.55–1.95			1.10
E. flabellatus	8.8.–10.2	2.1–2.4			1.10
E. flavoscutellaris	6.9	1.8			1.10
E. fuscicornis	8.8–10.8	2.1–2.4			1.15
E. luteolus	9.1–10.8	2.2–2.8			1.10
E. moluccanus	9.4	2.1			n.a.
E. nicricornis	10.2–11.5	2.9			1.00
E. nigriceps	7.6–9.8	1.7–1.8			1.10
E. nigroruber	10.7	2.5			1.03
E. pallidus	9.6–11.6	2.1–2.54			1.05
E. papuensis	9.4	2.35			1.13
E. pectinicornis	9.2–11.0	2.0–2.4			1.13
E. proximus	8.9–10.2	n.a.			1.15
E. robustus	10.8–11.7	2.6–2.9			1.14
E. sedlaceki	9.3–12.1	2.3–3.1			1.13
E. serratus (**b**)	8.9	2.25			1.40
E. slipinskii	7.2–8.4	1.65–1.9			0.75
E. subelectus	8.3–10.5	2.1–2.75			1.00
E. versicolor	8.3–11.1	2.0–2.85			1.20
E. wauensis	10.6–12.1	2.5–3.0			0.89

Table S5. Euclidian distances between *Eniclases* localities in kilometers. Aggregations designated as AGG1–4, see main text for further information.

		Sentani 275m	Elelim 580m	Domb 1150m	Bok1 1287m	Bok2 1250m	BokN 2100m	BokSW 1900m	Tikap 2150m	Yiwika 2100m
AGG1	Sentani	-								
AGG2	Elelim	189.3	-							
	Dombomi	205.8	22.1	-						
AGG3	Bokondini1	240.3	79.1	58.0	-					
AGG4	Bokondini2	240.4	79.4	58.3	0.3	-				
	Bokondini N	239.3	79.4	58.5	1.9	1.8	-			
	Bokondini SW	244.0	80.9	59.5	4.0	4.0	5.7	-		
	Tikapura	242.1	72.7	50.8	12.3	12.5	14.0	10.7	-	
	Yiwika	232.1	47.7	27.0	44.2	44.3	45.2	43.8	33.8	-
	Napua	252.1	64.7	46.5	54.6	54.7	56.1	52.7	42.1	21.4

Table S6. The occurrence of Metriorrhynchina species in the localities of central New Guinea.

Metriorrhynchini (non trichaline genera)

	S	D	Y	E	T	N	Bl	Bh	Sh
Metr. 1	-	-	-	-	-	-	-	+	1
Metr. 2	-	-	-	-	-	-	-	+	1
Metr. 3	-	-	-	-	-	-	-	+	1
Metr. 4	-	-	-	-	-	+	-	-	1
Metr. 5	-	-	+	-	-	-	-	-	1
Metr. 6	-	-	-	-	+	-	-	-	1
Metr. 7	-	-	-	-	+	-	-	-	1
Metr. 8	-	-	-	-	-	+	-	-	1
Metr. 9	-	-	-	-	+	-	-	-	1
Metr. 10	-	-	-	-	+	+	-	-	2
Metr. 11	-	-	+	-	-	-	-	+	2
Metr. 12	-	-	-	-	-	+	-	-	1
Metr. 13	+	-	-	-	-	-	-	-	1
Metr. 14	-	-	-	+	-	-	-	-	1
Metr. 15	-	-	-	-	+	-	-	-	1
Metr. 16	-	-	+	-	+	-	-	-	2
Metr. 17	-	+	-	-	-	-	-	-	1
Metr. 18	-	-	-	+	-	-	-	-	1
Metr. 19	-	-	-	-	-	-	-	+	1
Metr. 20	-	-	-	-	-	+	-	-	1
Metr. 21	-	-	-	-	-	+	-	-	1
Metr. 22	-	-	-	+	-	-	-	-	1
Metr. 23	-	-	-	+	-	-	-	-	1
Metr. 24	+	-	-	-	-	-	-	-	1
Metr. 25	-	-	-	-	-	-	-	+	1
Metr. 26	-	-	-	-	-	+	-	-	1
Metr. 27	-	-	-	-	+	-	-	+	2
Metr. 28	+	-	-	-	-	-	-	-	1
Metr. 29	+	-	-	-	-	-	+	-	2
Metr. 30	-	-	-	+	-	-	-	-	1
Metr. 31	-	-	-	-	-	-	-	+	1
Metr. 32	-	-	-	-	+	-	-	-	1
Metr. 33	-	-	-	-	-	+	-	-	1
Metr. 34	-	+	-	-	-	-	-	-	1
Metr. 35	-	-	+	-	-	-	-	-	1
Metr. 36	-	-	+	-	-	+	-	-	2
Metr. 37	-	-	-	-	-	-	-	+	1
Metr. 38	-	+	-	+	-	-	-	-	2
Metr. 39	-	-	-	+	-	-	-	-	1
Metr. 40	-	-	-	+	-	-	-	-	1
Metr. 41	-	+	+	-	-	-	-	+	3
Metr. 42	-	-	-	-	+	-	-	-	1
Metr. 43	-	-	-	+	-	-	-	-	1
Metr. 44	-	-	+	-	-	-	-	-	1
Metr. 45	+	-	-	-	-	-	-	+	2
Metr. 46	-	-	-	+	-	-	-	-	1
Metr. 47	+	-	-	-	-	-	-	-	1
Metr. 48	-	-	-	+	-	-	-	-	1
Metr. 49	-	-	-	+	-	-	-	-	1
Metr. 50	+	-	-	-	-	-	-	-	1
Metr. 51	-	-	+	-	-	-	-	-	1
Metr. 52	-	-	-	-	-	+	-	+	2
Metr. 53	-	-	-	-	+	-	-	+	2
Metr. 54	-	-	-	-	-	-	+	-	1
Metr. 55	-	-	-	-	-	+	-	-	1
Metr. 56	-	-	-	+	-	-	-	-	1
Metr. 57	-	-	+	-	-	-	-	-	1
Metr. 58	-	-	-	-	-	-	-	+	1
Metr. 59	-	-	+	-	-	-	-	-	1
Metr. 60	-	-	-	+	-	-	-	-	1
Metr. 61	-	-	-	-	-	+	-	-	1
Metr. 62	-	-	-	-	-	+	-	-	1
Metr. 63	-	-	+	-	-	-	-	-	1
Metr. 64	-	-	-	-	-	+	-	-	1
Metr. 65	-	+	-	-	-	-	-	-	1
Metr. 66	+	-	-	-	-	-	-	-	1
Metr. 67	-	-	-	-	-	-	+	-	1
Metr. 68	-	-	-	-	-	-	+	-	1
Metr. 69	-	-	-	+	-	-	-	-	1
Metr. 70	-	-	+	-	-	-	-	-	1
Metr. 71	-	-	-	-	-	+	-	-	1
Metr. 72	-	-	-	+	-	-	-	-	1
Metr. 73	-	-	+	-	-	-	-	-	1
Metr. 74	-	-	-	-	-	-	-	+	1
Metr. 75	-	+	-	+	-	-	-	-	2
Metr. 76	-	-	-	-	+	-	-	+	2
Metr. 77	-	-	+	-	+	-	-	-	2
Metr. 78	-	-	-	+	-	-	-	-	1
Metr. 79	-	-	-	+	-	-	-	-	1
Metr. 80	-	-	-	-	-	-	-	+	1
Metr. 81	-	-	-	-	-	+	-	+	2
Metr. 82	-	-	-	+	-	-	-	-	1
Metr. 83	-	-	-	+	-	-	-	-	1
Metr. 84	-	+	-	-	-	-	-	-	1
Metr. 85	-	-	+	-	-	-	-	-	1
Metr. 86	-	-	-	-	+	+	-	-	2
Metr. 87	-	-	+	-	-	-	-	-	1
Metr. 88	-	-	-	-	-	-	-	+	1
Metr. 89	+	-	-	-	-	-	-	-	1
Metr. 90	-	-	-	+	-	-	-	-	1
Metr. 91	-	+	-	-	-	-	-	-	1
Metr. 92	-	-	-	+	-	-	-	-	1
Metr. 93	-	+	-	-	-	-	-	-	1
Metr. 94	-	-	-	+	-	-	-	-	1
Metr. 95	-	-	-	-	-	+	-	-	1
Metr. 96	-	-	-	+	-	-	-	-	1
Metr. 97	-	-	-	+	-	-	-	-	1
Metr. 98	-	-	-	-	-	+	-	+	2
Metr. 99	-	-	-	-	-	-	-	+	1
Metr. 100	-	-	-	-	-	+	-	-	1
Metr. 101	-	-	-	-	-	-	-	+	1
Metr. 102	-	-	-	-	-	-	-	+	1
Metr. 103	-	-	-	-	-	-	+	-	1
Metr. 104	-	-	-	-	-	-	-	+	1
Metr. 105	-	-	-	-	-	-	-	+	1
Metr. 106	-	-	-	-	-	-	-	+	1

Metr. 107	-	-	-	+	-	-	-	-	1
Metr. 108	-	-	-	-	-	+	-	-	1
Metr. 109	-	-	-	-	-	+	-	-	1
Metr. 110	-	+	-	+	-	-	-	-	2
Metr. 111	-	-	-	+	-	-	-	-	1
Metr. 112	-	-	-	-	+	-	-	+	2
Metr. 113	-	-	+	-	-	-	-	-	1
Metr. 114	-	-	-	-	-	-	-	+	1
Metr. 115	-	-	-	+	-	-	-	-	1
Metr. 116	-	-	-	+	-	-	-	-	1
Metr. 117	-	-	-	+	-	-	-	-	1
Metr. 118	-	-	-	-	+	-	-	-	1
Metr. 119	-	-	-	-	+	-	-	-	1
Metr. 120	-	-	-	+	-	-	-	-	1
Metr. 121	+	-	-	-	-	-	-	-	1
Metr. 122	-	-	-	+	-	-	-	-	1
Metr. 123	-	-	+	-	-	-	-	+	2
Metr. 124	-	-	-	-	-	-	-	+	1
Metr. 125	-	-	-	-	-	-	-	+	1
Metr. 126	-	-	-	-	-	-	+	-	1
Metr. 127	-	-	-	+	-	-	-	-	1
Metr. 128	-	-	-	+	-	-	-	-	1
Metr. 129	-	-	-	-	+	-	-	-	1
Metr. 130	-	+	-	-	-	-	-	-	1
Metr. 131	-	-	-	-	-	+	-	-	1
Metr. 132	-	-	-	-	+	-	-	-	1
Metr. 133	-	-	-	+	-	-	+	-	2
Metr. 134	-	-	+	-	+	-	-	+	3
Metr. 135	-	-	-	-	-	-	+	-	1
Metr. 136	-	-	-	-	+	+	-	+	3
Metr. 137	-	-	+	-	-	-	-	-	1
Metr. 138	-	-	-	-	-	-	-	+	1
Metr. 139	-	-	-	-	+	-	-	-	1
Metr. 140	-	-	-	+	-	-	-	-	1
Metr. 141	-	-	+	-	-	+	-	-	2
Metr. 142	-	-	-	-	-	+	-	-	1
Metr. 143	-	-	-	-	-	+	-	-	1
Metr. 144	-	-	-	-	-	+	-	-	1
Metr. 145	-	-	-	-	+	-	-	-	1
Metr. 146	-	+	-	-	-	-	-	+	2
Metr. 147	-	-	-	+	-	-	-	-	1
Metr. 148	-	-	-	-	+	-	-	-	1
Metr. 149	-	-	-	+	-	-	-	-	1
Metr. 150	-	-	+	-	-	+	-	-	2
Metr. 151	-	-	+	-	+	+	-	-	3
Metr. 152	-	-	-	+	-	-	+	-	2
Metr. 153	-	-	+	-	-	-	-	-	1
Metr. 154	-	-	-	-	+	-	-	+	2
Metr. 155	-	-	-	+	-	-	-	-	1
Metr. 156	+	-	-	-	-	-	-	-	1
Metr. 157	-	-	-	+	-	-	-	-	1
Metr. 158	-	-	-	+	-	-	-	-	1
Metr. 159	-	-	-	-	+	-	-	-	1
Metr. 160	-	-	-	-	+	+	-	+	3
Metr. 161	-	-	+	-	-	-	-	+	2
Metr. 162	-	-	-	-	-	-	-	+	1
Metr. 163	-	-	-	-	-	-	-	+	1
Metr. 164	-	-	-	-	+	-	-	-	1
Metr. 165	-	-	-	-	-	-	-	+	1
Metr. 166	-	-	-	-	-	+	-	-	1
Metr. 167	-	-	-	-	-	-	+	-	1
Metr. 168	-	-	-	+	-	-	-	-	1
Metr. 169	+	-	-	+	-	-	-	-	2
Metr. 170	-	-	-	-	-	-	+	-	1
Metr. 171	-	-	-	-	+	+	-	-	2
Metr. 172	-	+	-	+	-	-	-	-	2
Metr. 173	-	-	-	-	-	-	-	+	1
Metr. 174	-	-	+	-	-	-	-	-	1
Metr. 175	-	-	-	-	+	-	-	-	1
Metr. 176	-	-	-	-	-	-	-	+	1
Metr. 177	-	-	-	-	-	-	-	+	1
Metr. 178	-	-	+	-	-	+	-	-	2
Metr. 179	-	-	-	+	-	-	-	-	1
Metr. 180	-	-	-	+	-	-	-	-	1
Metr. 181	-	-	+	-	-	-	-	-	1
Metr. 182	-	-	-	-	-	-	-	+	1
Metr. 183	-	-	+	-	+	+	-	+	4
Metr. 184	-	-	-	-	-	-	-	+	1
Metr. 185	-	-	-	+	-	-	+	-	2
Metr. 186	-	-	-	+	-	-	-	-	1
Metr. 187	-	-	-	+	-	-	-	-	1
Metr. 188	-	-	-	+	-	-	-	-	1
Metr. 189	-	-	-	-	-	-	-	+	1
Metr. 190	-	-	-	-	+	-	-	-	1
Metr. 191	+	-	-	-	-	-	-	-	1
Metr. 192	-	-	-	+	-	-	-	-	1
Metr. 193	+	-	-	-	-	-	-	-	1
Metr. 194	-	+	-	+	-	-	+	-	3
Metr. 195	-	-	-	-	+	-	-	-	1
Metr. 196	-	+	-	-	-	-	-	-	1
Metr. 197	-	+	+	-	-	-	-	-	2
Metr. 198	-	-	-	+	-	-	-	-	1
Metr. 199	-	+	-	-	-	-	-	-	1
Metr. 200	-	-	-	-	+	+	-	-	2
Metr. 201	-	-	-	-	+	-	-	-	1
Metr. 202	-	-	-	+	-	-	-	-	1
Metr. 203	-	-	-	-	-	+	-	-	1
Metr. 204	-	-	-	-	+	-	-	+	2
Metr. 205	-	-	-	-	-	+	-	-	1
Metr. 206	+	-	-	-	-	-	-	-	1
Metr. 207	-	+	-	-	-	-	-	-	1
Metr. 208	-	-	+	-	-	+	-	-	2
Metr. 209	-	-	-	+	-	-	+	-	2
Metr. 210	+	-	-	+	-	-	-	-	2
Metr. 211	-	-	-	+	-	-	-	+	2
Metr. 212	-	+	-	+	-	-	-	-	2
Metr. 213	-	-	-	+	-	-	+	-	2
Metr. 214	-	-	-	+	-	-	-	-	1
Metr. 215	-	-	-	-	+	-	-	-	1
Metr. 216	-	+	-	-	-	-	-	-	1
Metr. 217	-	+	-	+	-	-	-	-	2
Metr. 218	-	+	-	-	-	-	-	-	1
Metr. 219	-	-	-	-	-	-	+	-	1
Metr. 220	-	-	-	+	-	-	-	-	1
Metr. 221	-	-	-	-	+	-	-	-	1
Metr. 222	-	-	-	+	-	-	-	-	1
Metr. 223	-	-	-	-	-	-	-	+	1
Metr. 224	-	-	-	-	-	+	-	-	1

Metr. 225	-	-	-	-	+	-	-	-	1
Metr. 226	-	-	-	+	-	-	-	-	1
Metr. 227	-	-	+	-	-	+	-	+	3
Metr. 228	+	+	-	+	-	-	-	-	3
Metr. 229	-	-	-	+	-	-	-	-	1
Metr. 230	-	-	+	-	-	-	-	-	1
Metr. 231	-	-	-	-	-	-	-	+	1
Metr. 232	-	-	-	+	-	-	-	-	1
Metr. 233	-	-	-	-	-	-	-	+	1
Metr. 234	-	-	-	+	-	-	-	-	1
Metr. 235	-	-	+	-	-	-	-	-	1
Metr. 236	-	-	-	-	-	+	-	-	1
Metr. 237	-	-	-	+	-	-	-	-	1
Metr. 238	-	+	-	-	-	-	-	-	1
Metr. 239	+	-	-	-	-	-	-	-	1
Metr. 240	-	-	-	+	-	-	-	-	1
Metr. 241	-	-	-	-	-	+	-	-	1
Metr. 242	-	-	-	-	-	-	+	-	1
Metr. 243	+	-	-	-	-	-	-	-	1
Metr. 244	-	-	+	-	-	-	-	-	1
Metr. 245	-	-	-	-	+	+	-	+	3
Total	19	24	35	73	40	44	17	55	

Trichalini (without *Eniclases*)

Trich. 1	-	-	-	+	-	-	-	-	1
Trich. 2	-	-	+	-	+	+	-	-	3
Trich. 3	-	-	-	-	-	-	+	-	1
Trich. 4	-	-	-	+	-	-	-	-	1
Trich. 5	+	-	-	-	-	-	-	-	1
Trich. 6	-	-	+	-	-	-	-	-	1
Trich. 7	-	-	+	-	-	-	-	-	1
Trich. 8	-	-	-	-	-	-	+	-	1
Trich. 9	+	-	-	+	-	-	-	-	2
Trich. 10	-	-	-	-	-	-	+	-	1
Trich. 11	-	-	+	-	-	-	-	-	1
Trich. 12	-	-	-	-	+	-	-	-	1
Trich. 13	-	-	-	-	+	-	-	-	1
Trich. 14	-	-	-	+	-	-	-	-	1
Trich. 15	-	-	-	+	+	-	-	-	2
Trich. 16	+	-	-	-	-	-	-	-	1
Trich. 17	-	-	-	+	-	-	-	-	1
Trich. 18	-	-	+	-	+	-	-	-	2
Trich. 19	+	-	-	-	-	-	-	-	1
Trich. 20	-	-	-	-	+	-	-	-	1
Trich. 21	-	-	-	-	+	-	-	-	1
Trich. 22	-	-	-	-	+	-	-	-	1
Trich. 23	+	-	-	-	-	-	-	-	1
Trich. 24	-	-	-	-	-	+	-	-	1
Trich. 25	-	-	-	-	+	-	-	-	1
Trich. 26	-	-	-	+	-	-	+	-	2
Trich. 27	-	-	-	+	-	-	-	-	1
Trich. 28	-	-	-	+	-	-	-	-	1
Trich. 29	-	-	-	-	+	+	-	-	2
Trich. 30	-	-	-	+	-	-	-	-	1
Trich. 31	-	-	-	+	-	-	+	-	2
Trich. 32	-	-	-	-	+	-	+	-	2
Trich. 33	+	-	-	-	-	-	-	-	1
Trich. 34	-	-	-	-	-	-	+	-	1
Trich. 35	-	-	+	-	+	-	-	-	2
Trich. 36	+	-	-	-	-	-	-	-	1
Trich. 37	-	-	+	+	+	+	-	-	4
Trich. 38	-	-	-	-	-	-	-	+	1
Total	7	0	7	12	13	4	7	1	

Eniclases species

	S	D	Y	E	T	N	Bl	Bh	Sh
Enic. 1	+	-	-	-	-	-	-	-	1
Enic. 2	-	-	-	+	-	-	-	-	1
Enic. 3	-	-	-	-	-	-	-	+	1
Enic. 4	+	-	-	-	-	-	-	-	1
Enic. 5	+	-	-	+	-	-	-	-	2
Enic. 6	-	+	-	+	-	-	+	-	3
Enic. 7	-	-	-	+	-	-	-	-	1
Enic. 8	-	-	-	+	-	-	+	-	2
Enic. 9	+	-	-	-	-	-	-	-	1
Enic. 10	+	-	-	-	-	-	-	-	1
Enic. 11	-	-	+	-	+	-	-	+	1
Enic. 12	-	-	-	+	-	-	-	-	1
Total	5	1	1	6	1	0	2	2	

Abbreviations:

S – Sentani, D –Dombomi,
Y – Yiwika, E – Elelim,
T – Tikapura,N – Napua,
Bl – Bokondini 1250 m
Bh –Bokondini 2000 m
Sh –the number of localities which share the species

+ – record in the locality
- – absent in the locality

Enic. 1 – *Eniclases apertus*
Enic. 2 – *Eniclases bicolor*
Enic. 3 – *Eniclases bokondinensis*
Enic. 4 – Eniclases *brancuccii*
Enic. 5 – *Eniclases divaricatus*
Enic. 6 – *Eniclases elelimensis*
Enic. 7 – *Eniclases infuscatus*
Enic. 8 – *Eniclases niger*
Enic. 9 – *Eniclases similis*
Enic. 10 – *Eniclases* sp. B
Enic. 11 – *Eniclases tikapurensis*
Enic. 12 – *Eniclases variabilis*

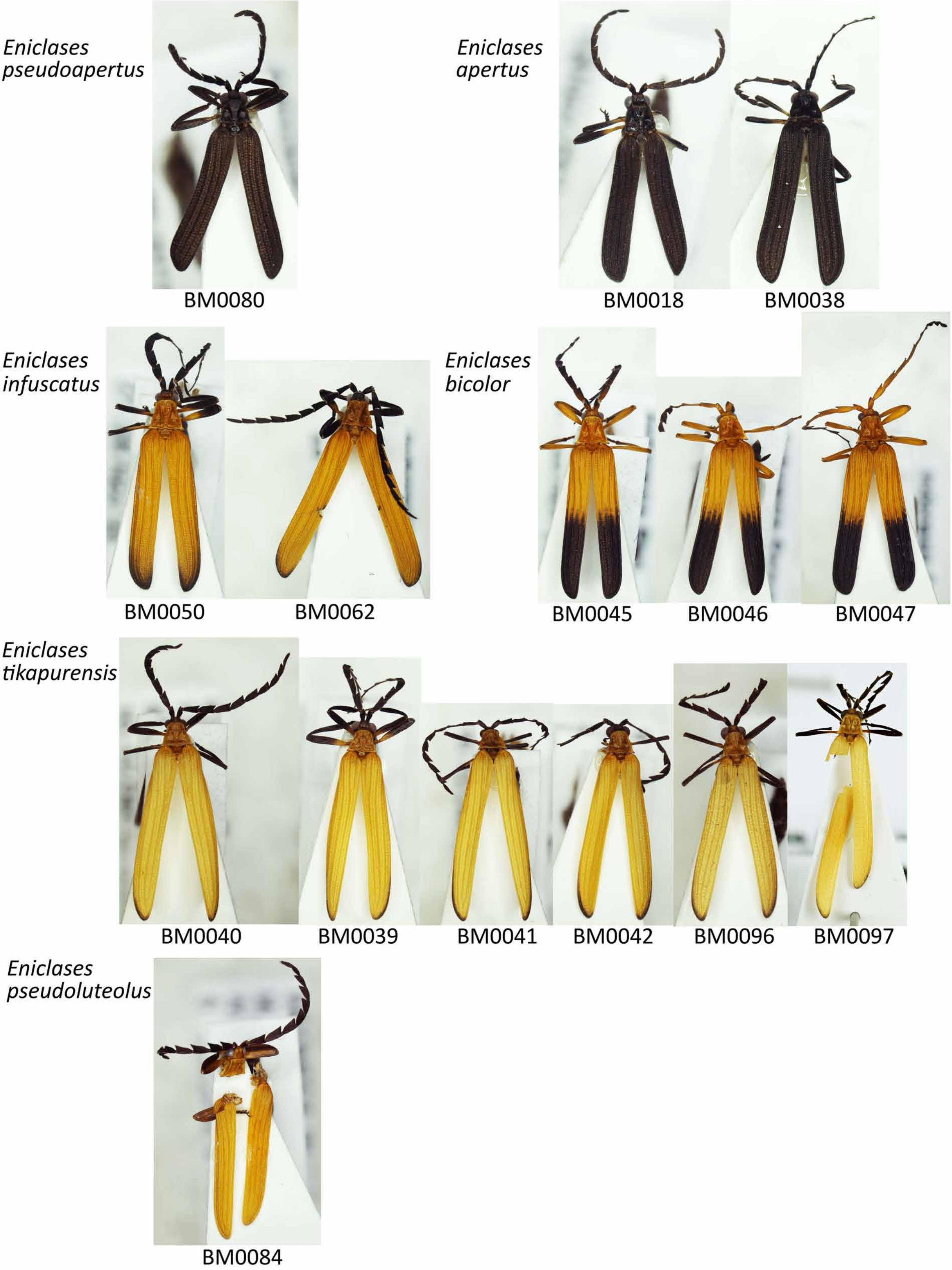

Supplementary figure S1. Aposematic patterns of sequenced specimens from New Guinea (part 1).

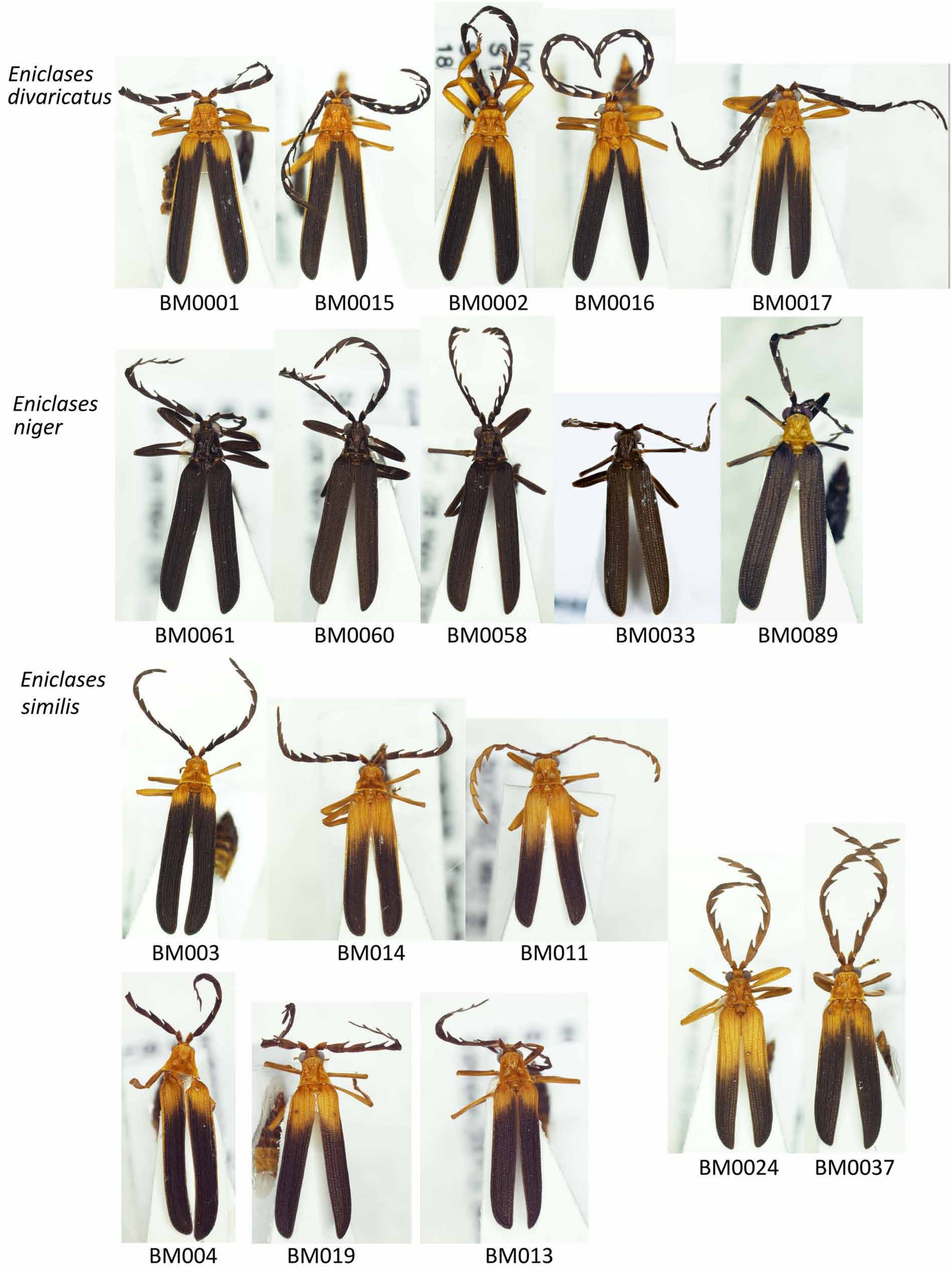

Supplementary figure S2. Aposematic patterns of sequenced specimens from New Guinea (part 2).

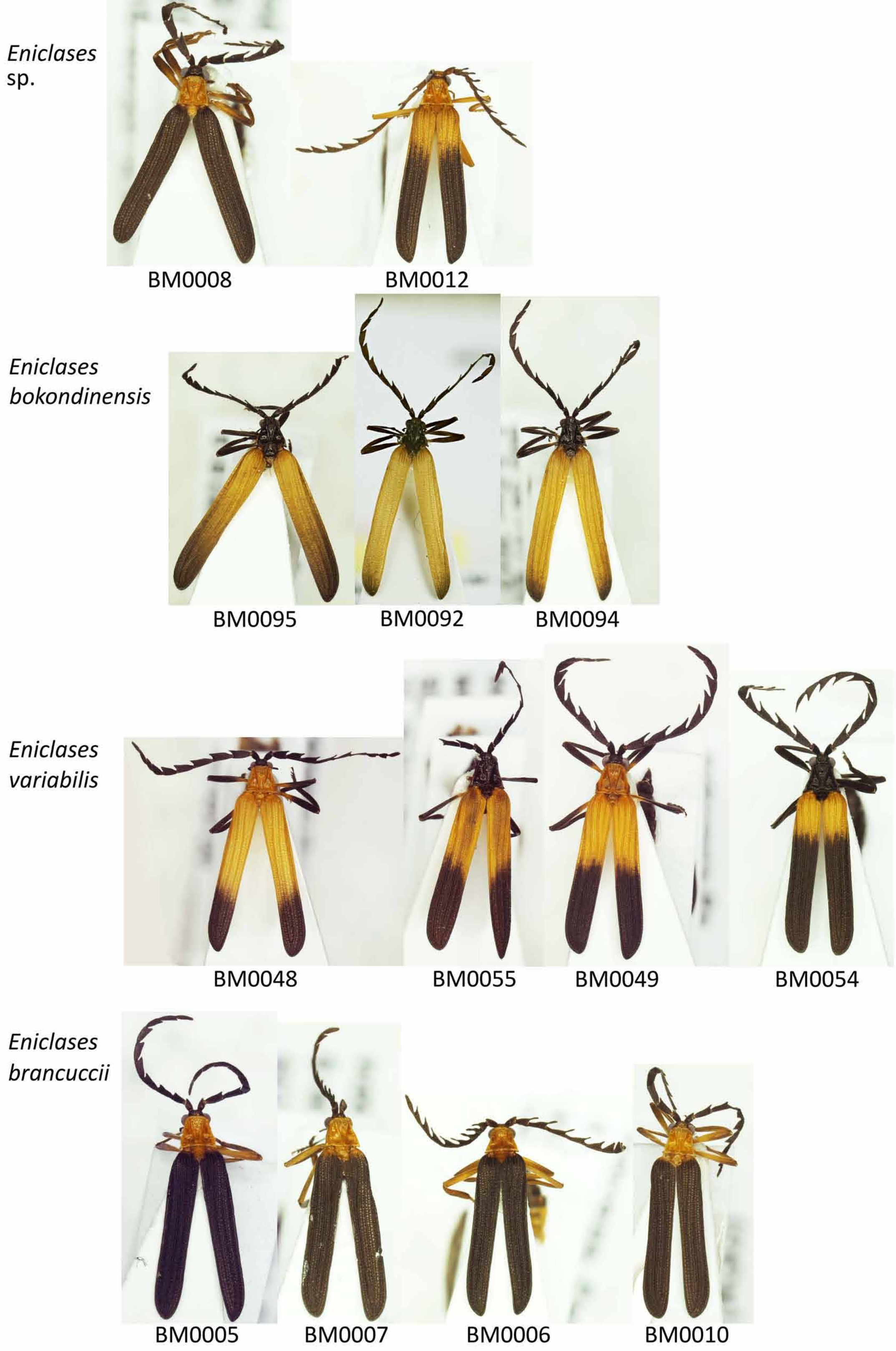

Supplementary figure S3. Aposematic patterns of sequenced specimens from New Guinea (part 3).

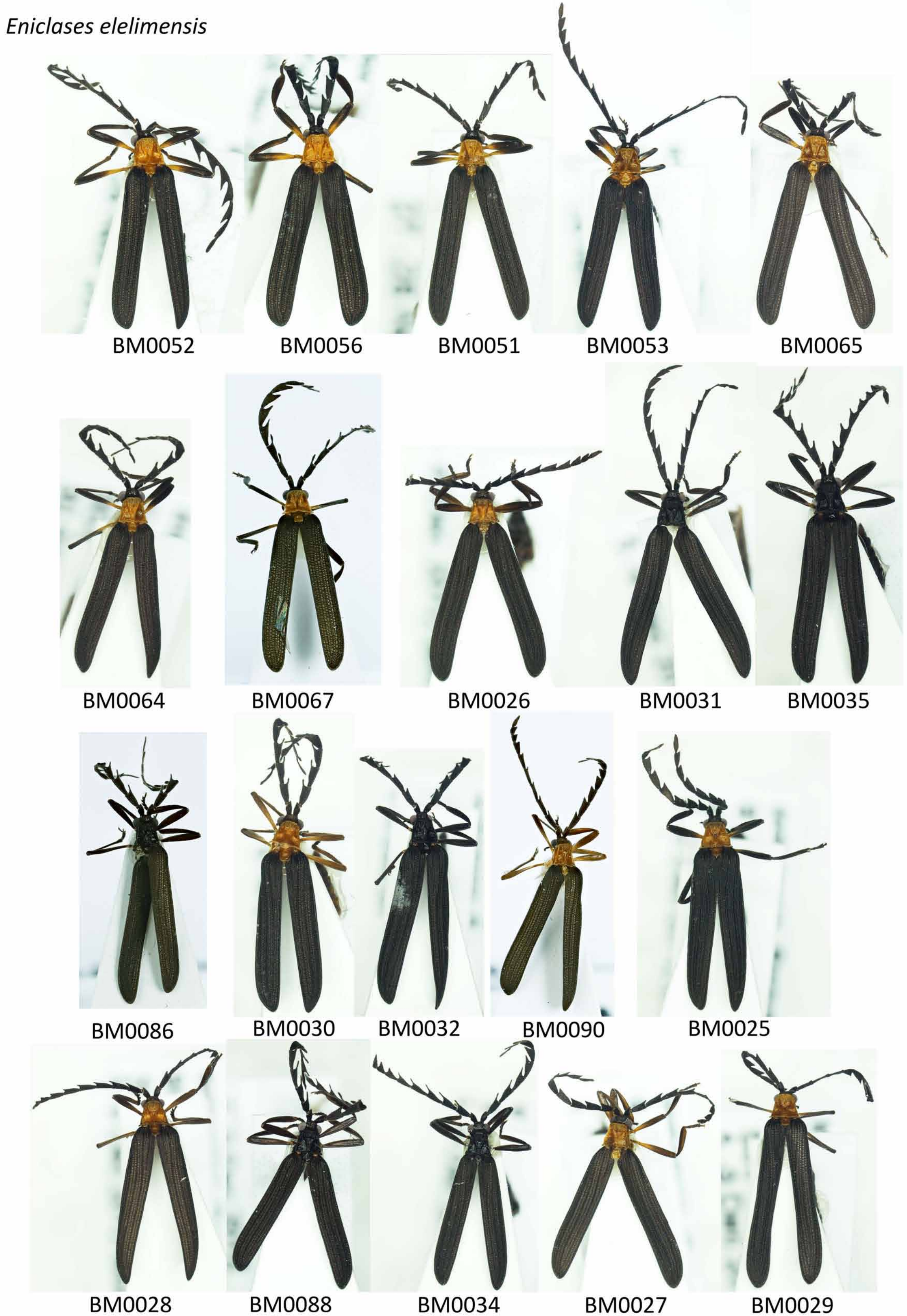

Supplementary figure S4. Aposematic patterns of sequenced specimens from northern New Guinea (part 4).

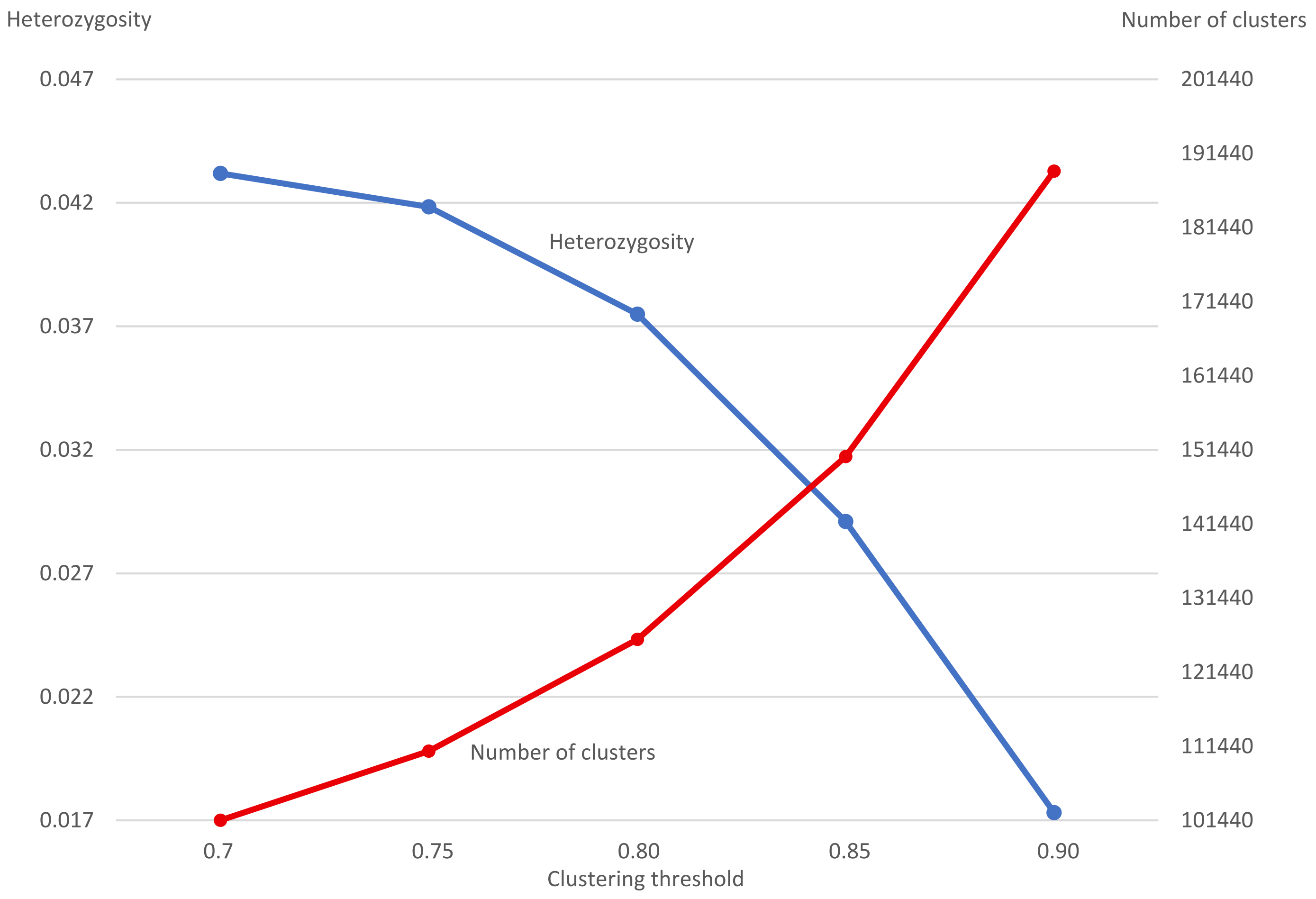

Supplementary Fig. S5 Testing the effects of clustering threshold on the individual heterozygosity and numbers of clusters produced.

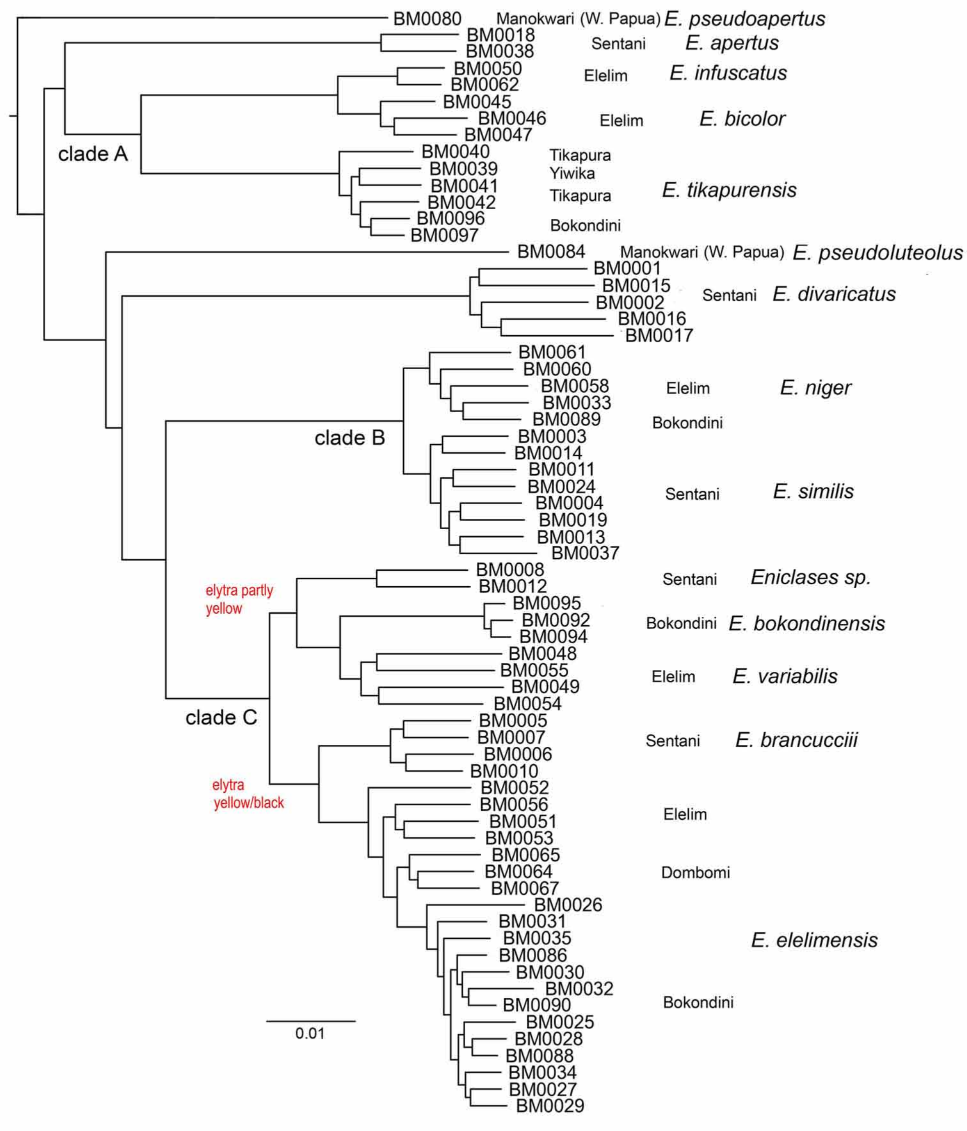

Supplementary Figure S6. Full length RAD-seq tree.

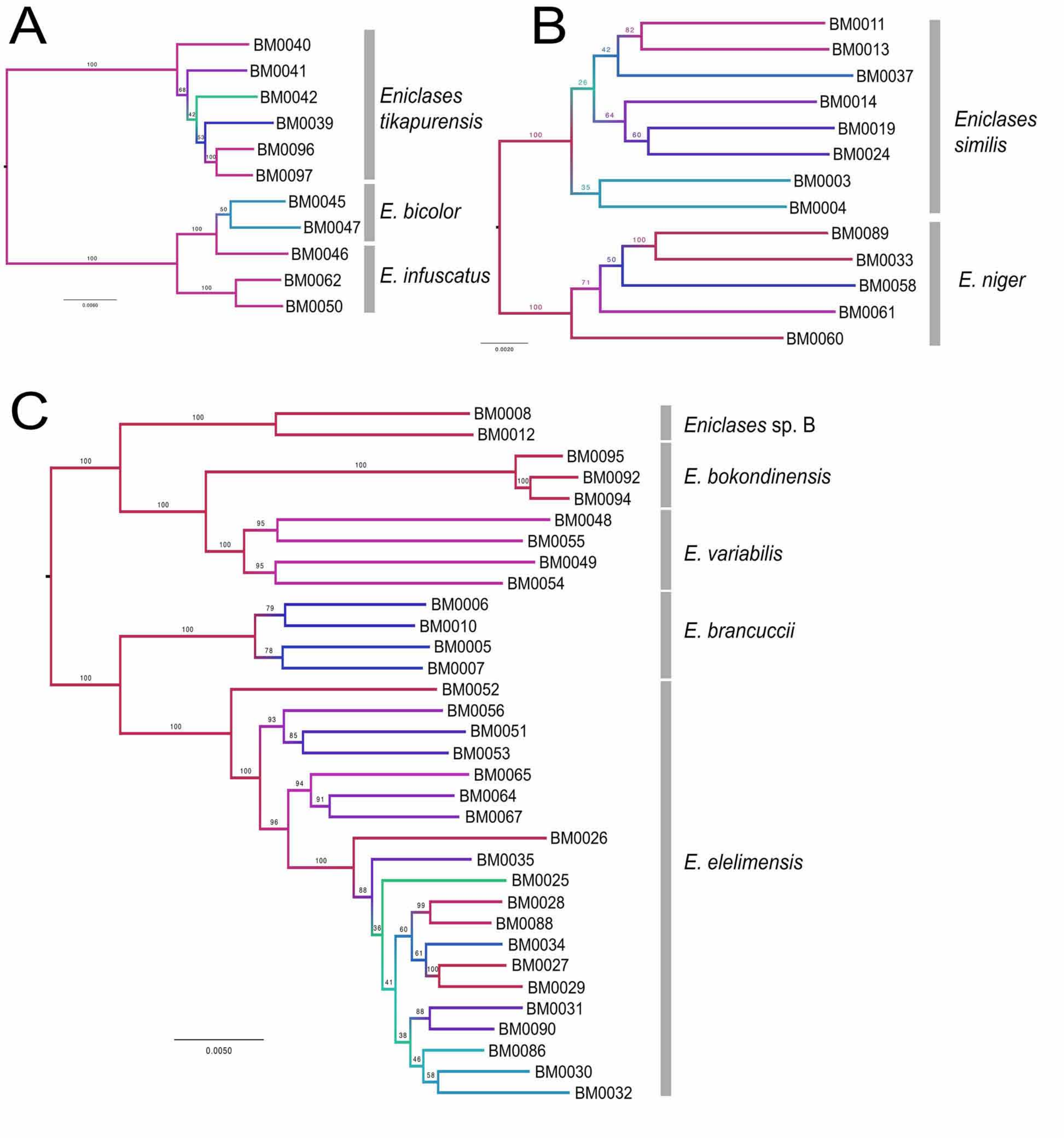

Supplementary figure S7. The full resolution RAD-based trees for individual subclades based on subset read filtering. The datasets were filtered using the same settings as for the whole dataset analysis (MinCov=4, MinDepth=5, Wclust=0.85).

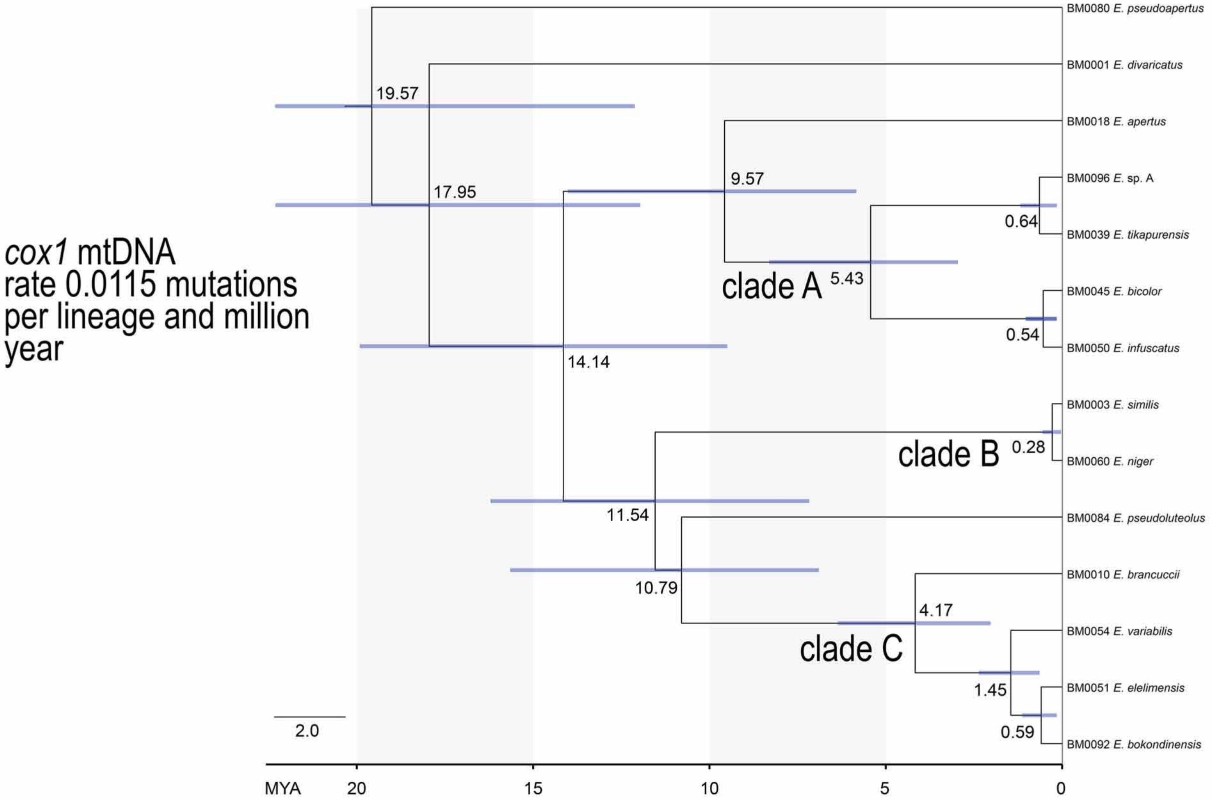

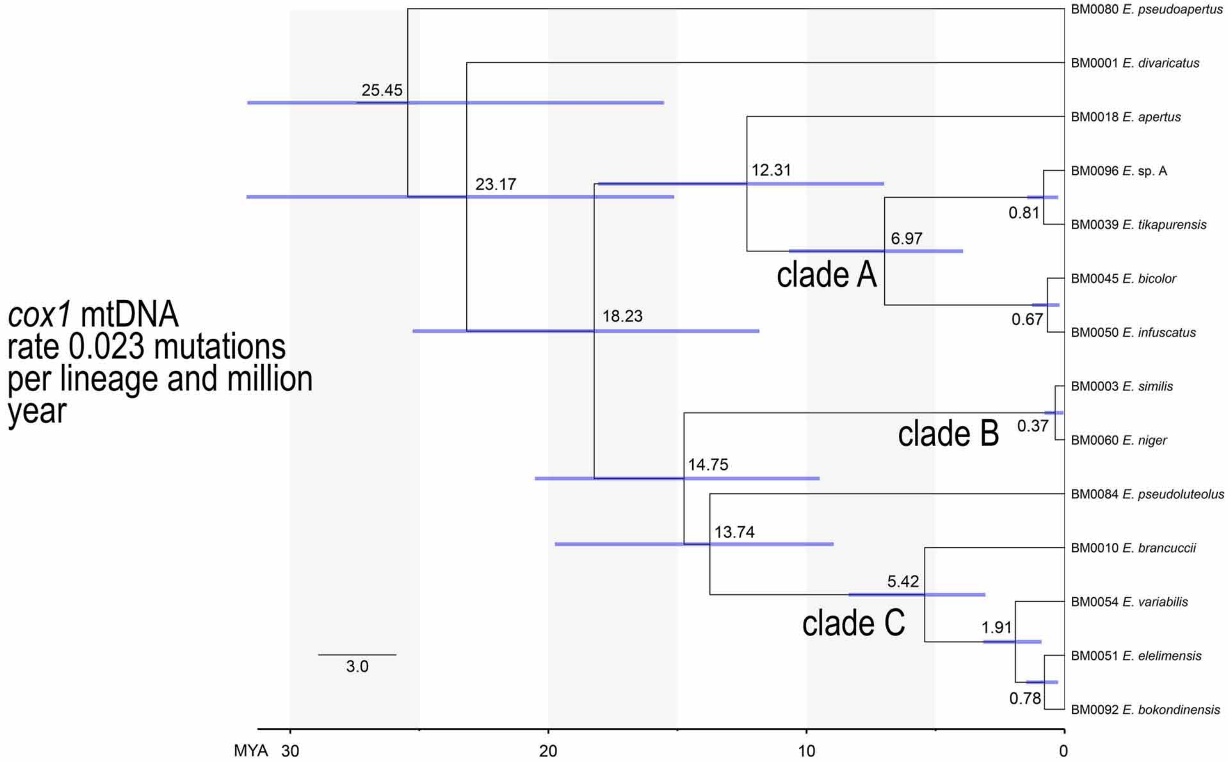

Supplementary figure S8. The dated trees based on *cox1* mtDNA dataset using two different mutation rates.

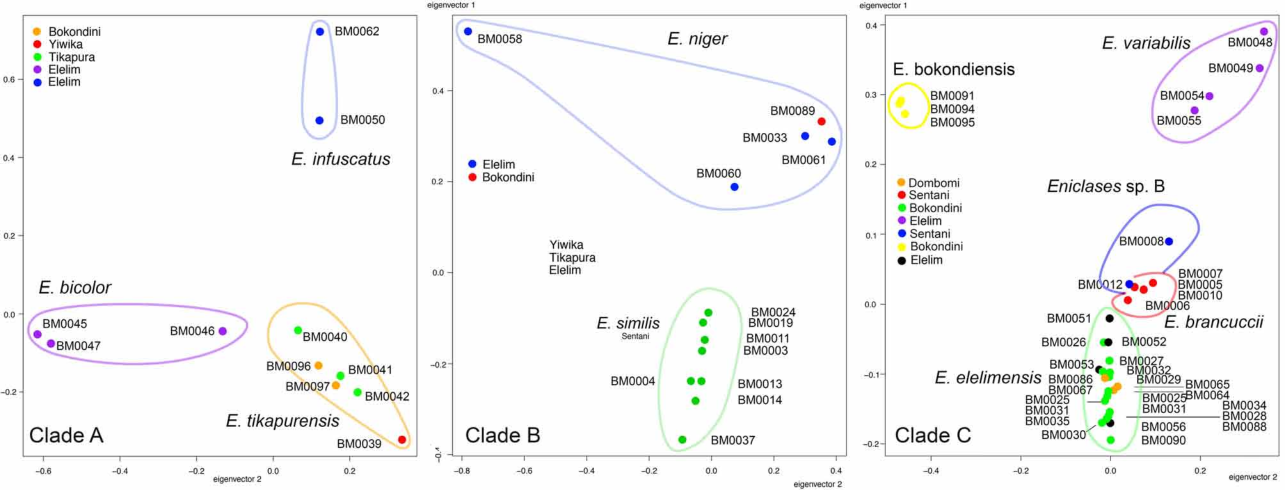

Supplementary figure S9. The principal component analysis (PCA) of the clades A, B, C inferred from RAD dataset.

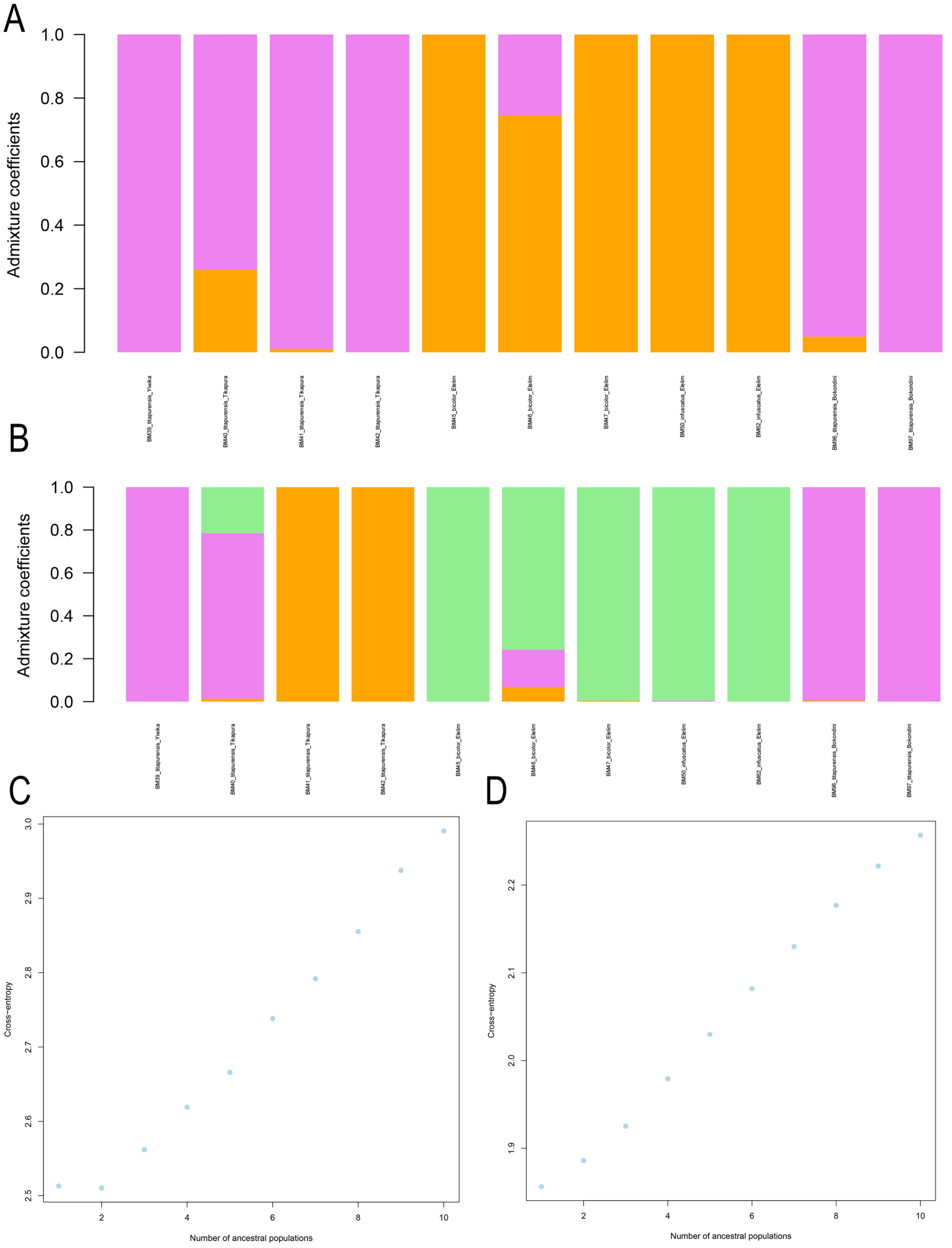

Supplementary Fig. S10 Plots of inferred individuals admixture coefficients based on sparse non-negative matrix factorization (sNMF) implemented in R package LEA for the clade A as defined by the phylogenetic analyses.The figures show the following K genetic clusters: A, K=2; B, K=3; C, cross entropy graph for the clade A; D, cross entrophy graph for the clade B.

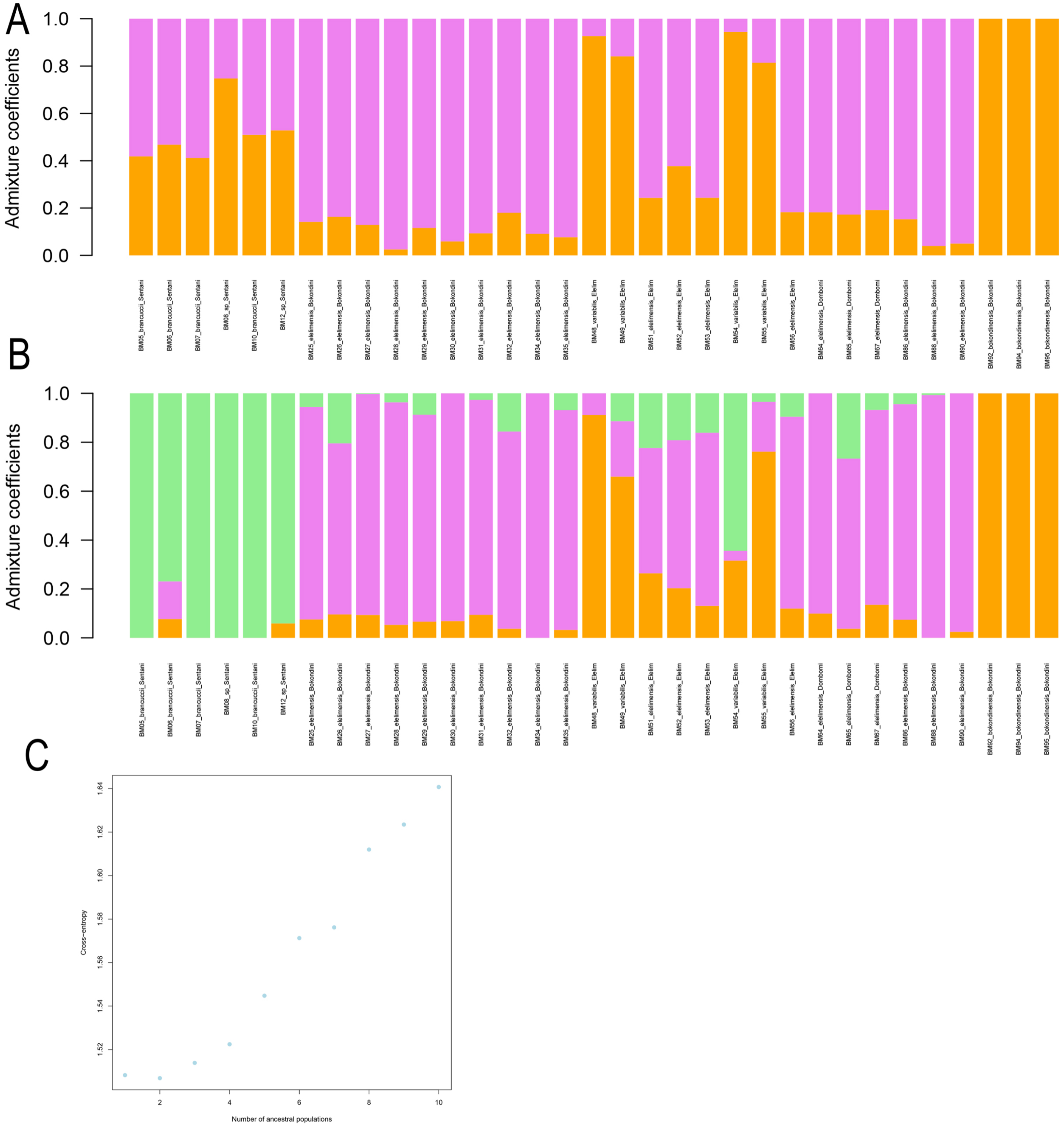

Supplementary Fig. S11 Plots of inferred individuals admixture coefficients based on sparse non-negative matrix factorization (sNMF) implemented in R package LEA for the clade C as defined by the phylogenetic analyses. The figures show the following K genetic clusters: A, K=2; B, K=3; C, cross entropy graph for the clade C.

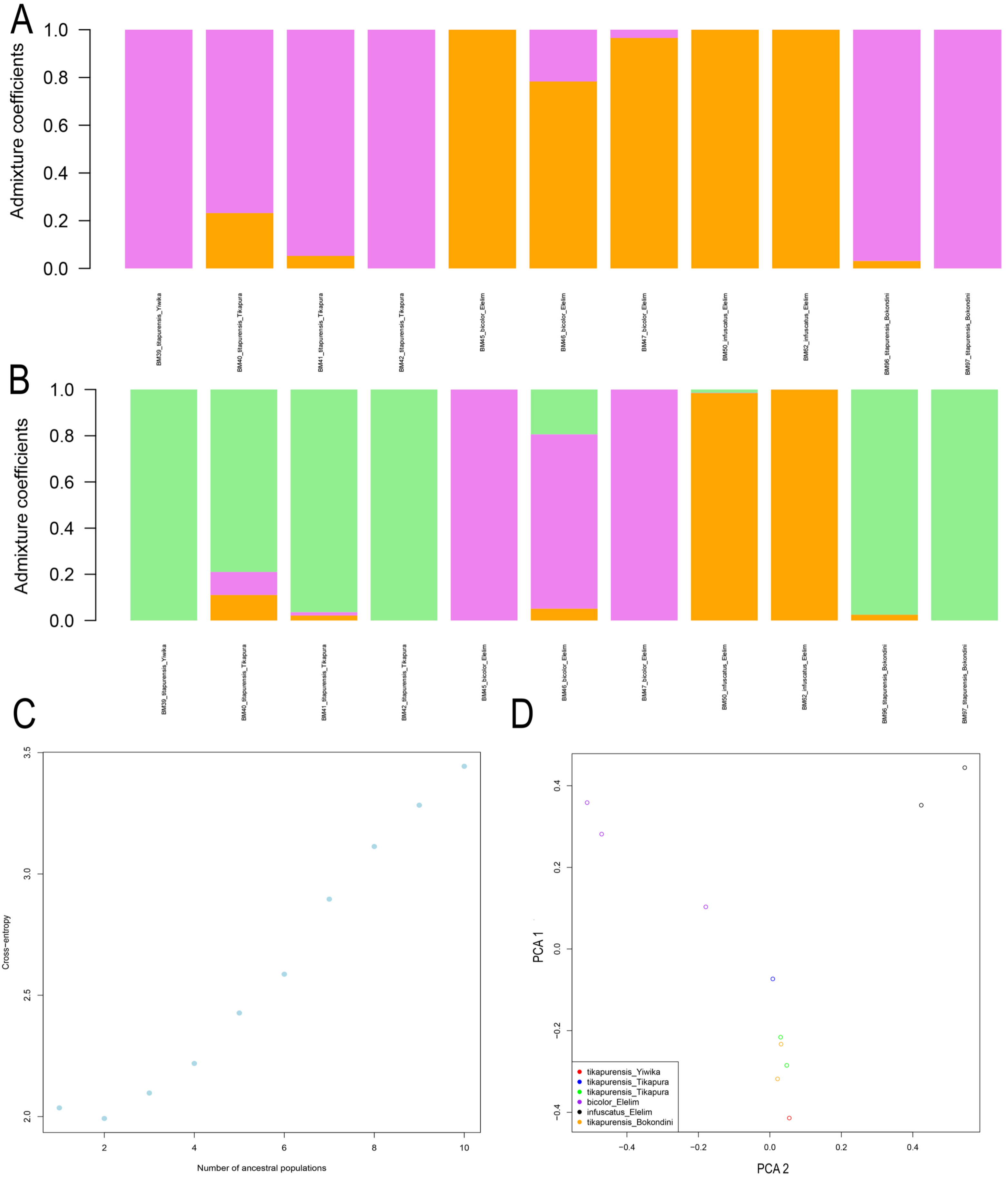

Supplementary Fig. S12 Plots of inferred individuals admixture coefficients based on sparse non-negative matrix factorization (sNMF) implemented in R package LEA for the clade A as defined by the phylogenetic analyses. The figures show the following K genetic clusters: A, K=2; B, K=3; C, cross entropy graph for the clade A; D, distribution of Eniclases individuals from the clade A along principal component (PC) scores (PC1 17.2%; PC2 14.3%) of genetic variation based on the analysis of the RAD dataset. Both analyses based on the iPYRAD dataset calculated from individuals of the clade A only.

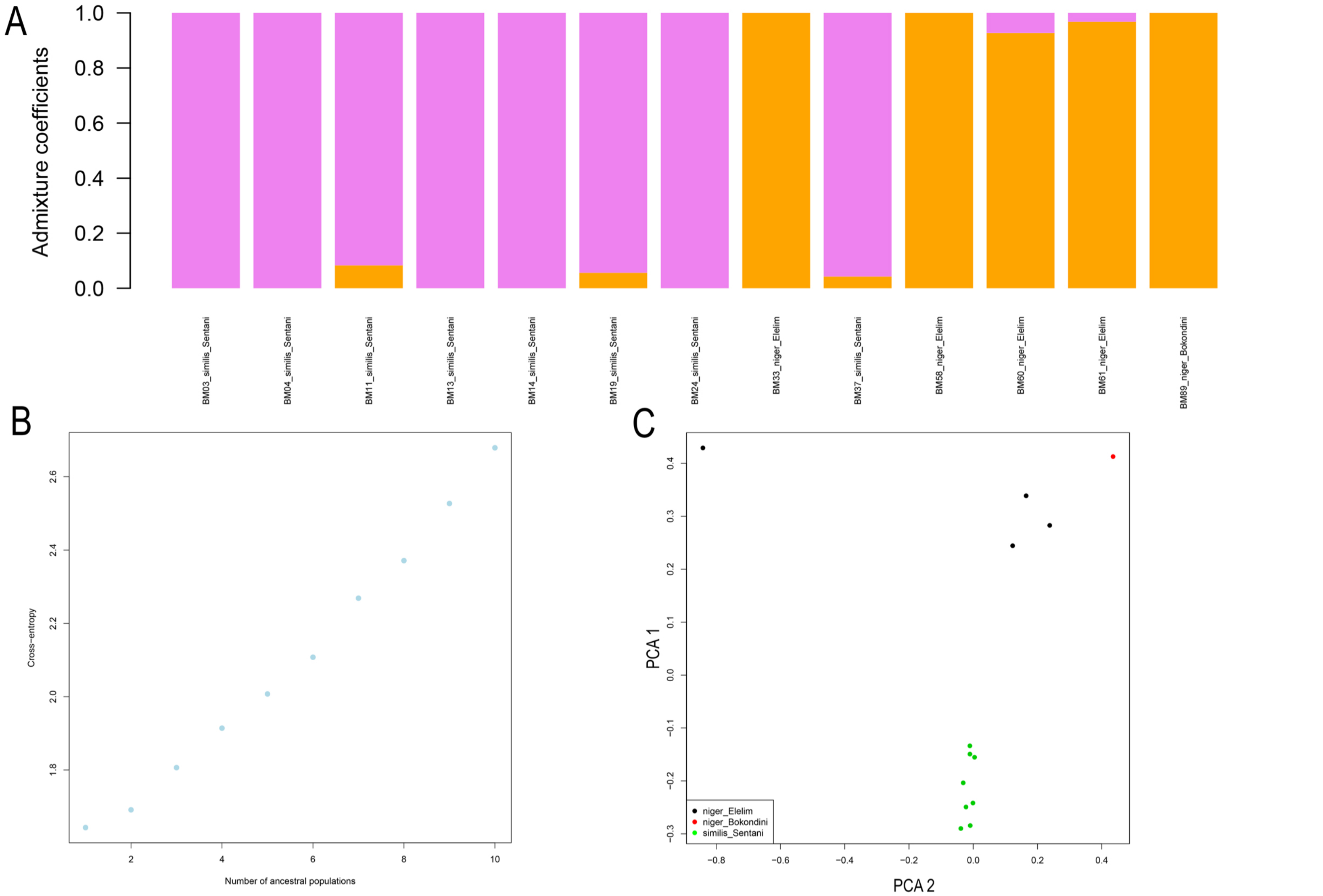

Supplementary Fig. S13 Plot of inferred individuals admixture coefficients based on sparse non-negative matrix factorization (sNMF) implemented in R package LEA for the clade B as defined by the phylogenetic analyses. The figure show the following K genetic cluster: A, K=2; B, cross entropy graph for the clade B; C, distribution of Eniclases individuals from the clade B along principal component (PC) scores (PC1 12.4%; PC2 9.6%) of genetic variation based on the analysis of the RAD dataset. Both analyses based on the iPYRAD dataset calculated from individuals of the clade B only.

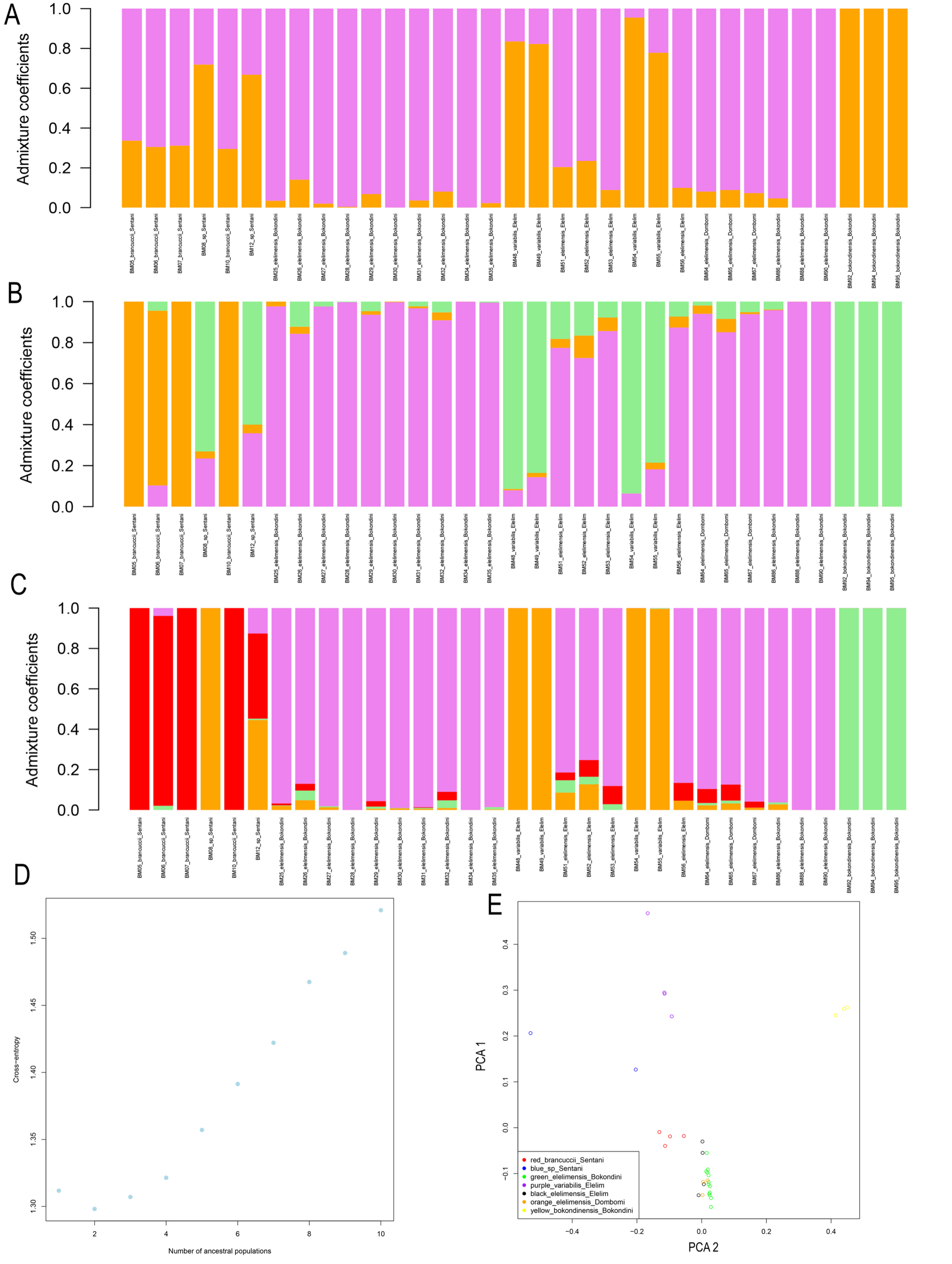

Supplementary Fig. S14 Plots of inferred individuals admixture coefficients based on sparse non-negative matrix factorization (sNMF) implemented in R package LEA for the clade C as defined by the phylogenetic analyses. The figures show the following K genetic clusters: A, K=2; B, K=3; C, K=4; D, cross entropy graph for the clade C; E, distribution of Eniclases individuals from the clade C along principal component (PC) scores (PC1 6.1%; PC2 5.6%) of genetic variation based on the analysis of the RAD dataset. Both analyses based on the iPYRAD dataset calculated from individuals of the clade C only.
